# Sonic Hedgehog acts as a macrophage chemoattractant during regeneration of the gastric epithelium

**DOI:** 10.1038/s41536-021-00196-2

**Published:** 2022-01-12

**Authors:** Jayati Chakrabarti, Martha Dua-Awereh, Michael Schumacher, Amy Engevik, Jennifer Hawkins, Michael A. Helmrath, Yana Zavros

**Affiliations:** 1 Department of Cellular and Molecular Medicine, University of Arizona College of Medicine, Tucson, AZ USA; 2 Department of Pharmacology and Systems Physiology, University of Cincinnati, Cincinnati, OH USA; 3 Division of Gastroenterology, Children’s Hospital Los Angeles, Los Angeles, CA USA; 4 Department of Regenerative Medicine and Cell Biology, Medical University of South Carolina, Charleston, SC USA; 5 Department of Pediatric Surgery, Cincinnati Children’s Hospital Medical Center, Cincinnati, OH USA

**Keywords:** Physiology, Ulcers

## Abstract

Sonic Hedgehog (Shh), secreted from gastric parietal cells, contributes to the regeneration of the epithelium. The recruitment of macrophages plays a central role in the regenerative process. The mechanism that regulates macrophage recruitment in response to gastric injury is largely unknown. Here we tested the hypothesis that Shh stimulates macrophage chemotaxis to the injured epithelium and contributes to gastric regeneration. A mouse model expressing a myeloid cell-specific deletion of Smoothened (LysMcre/+;Smof/f) was generated using transgenic mice bearing loxP sites flanking the Smo gene (Smo loxP) and mice expressing a Cre recombinase transgene from the Lysozyme M locus (LysMCre). Acetic acid injury was induced in the stomachs of both control and LysMcre/+;Smof/f (SmoKO) mice and gastric epithelial regeneration and macrophage recruitment analyzed over a period of 7 days post-injury. Bone marrow-derived macrophages (BM-Mø) were collected from control and SmoKO mice. Human-derived gastric organoid/macrophage co-cultures were established, and macrophage chemotaxis measured. Compared to control mice, SmoKO animals exhibited inhibition of ulcer repair and normal epithelial regeneration, which correlated with decreased macrophage infiltration at the site of injury. Bone marrow chimera experiments using SmoKO donor cells showed that control chimera mice transplanted with SmoKO bone marrow donor cells exhibited a loss of ulcer repair, and transplantation of control bone marrow donor cells to SmoKO mice rescued epithelial cell regeneration. Histamine-stimulated Shh secretion in human organoid/macrophage co-cultures resulted in macrophage migration toward the gastric epithelium, a response that was blocked with Smo inhibitor Vismodegib. Shh-induced macrophage migration was mediated by AKT signaling. In conclusion, Shh signaling acts as a macrophage chemoattractant via a Smo-dependent mechanism during gastric epithelial regeneration in response to injury.

## Introduction

Sonic Hedgehog (Shh) is expressed in a number of adult organs including the stomach. Shh signaling plays a fundamental role in the regulation of (1) normal epithelial cell differentiation and proliferation, (2) tissue homeostasis, (3) the gastric immune response to *Helicobacter pylori* (*H. pylori*) infection, and (4) regeneration within the adult stomach^1–7^. Our study seeks to identify the mechanism by which the Hedgehog (Hh)-signaling pathway regulates the regeneration of the gastric epithelium in response to injury^7,8^.

The immune response is crucial for repair in tissues that include the kidney^9,10^, skin^11^, and stomach^12^. In particular, macrophages are key immune cells that secrete necessary cytokines, chemokines, and pro-angiogenic factors that are necessary regulators for repair^13^. We have documented that Shh may act as a macrophage chemoattractant in response to infection and injury^7,8^. Previous work supports the concept that Shh acts as a macrophage chemoattractant as mice expressing a tamoxifen-inducible parietal cell-specific deletion of Shh exhibit decreased macrophage infiltration and neovascularization that coincides with failure to repair and regenerate the gastric epithelium in response to injury^8^. Another study by our group using bone marrow chimera experiments with donor cells collected from mice that have a myeloid cell-specific deletion of the Hh signal transduction protein smoothened (LysMcre/+;Smof/f), demonstrated that Shh signals to the macrophages to initiate recruitment during the initiation of gastritis in response to *H. pylori*^14^. The current study extends our previous research by identifying the role of Shh as a macrophage chemoattractant during repair using the LysMcre/+;Smof/f (SmoKO) mouse model and extends this to human tissues using human-derived organoid/macrophage co-cultures.

## Results

### LysMcre/+;Smof/f (SmoKO) mice exhibit disrupted normal gastric epithelial regeneration

**Fig. 1:**
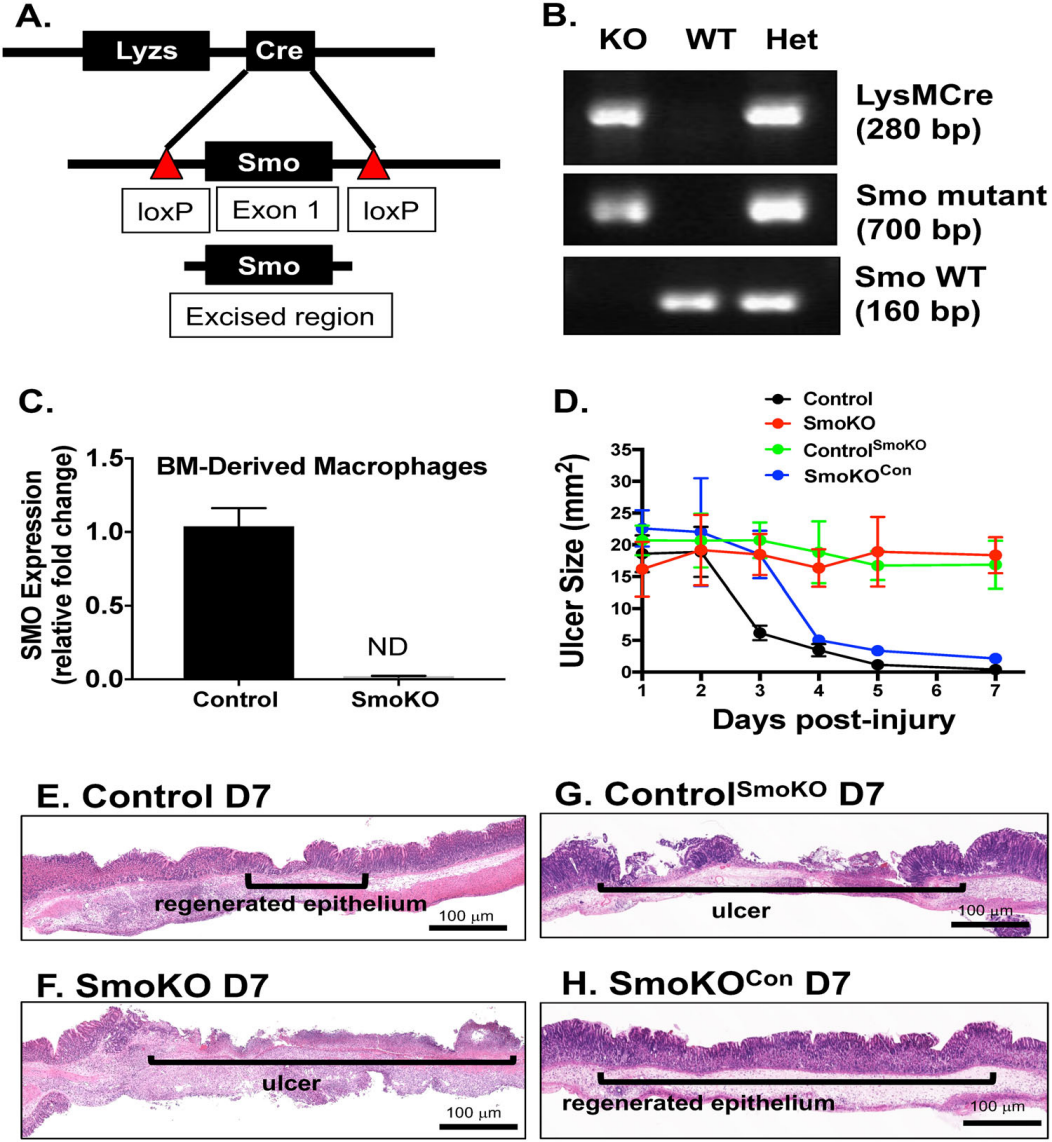
Generation of LysMCre/SmoKO mice. **A** The LysMCre/SmoKO mice were generated by crossing the LysMCre with the Smoflx/flx. The LysMCre knock-in allele has a nuclear-localized Cre recombinase inserted into the lysozyme 2 gene (Lyz2). When crossed with Smoflx/flx mice, that have loxP sites flanking exon 1 of the Smo gene, Cre-mediated recombination results in deletion of the targeted gene in the myeloid cell lineage. Deletion of Smo was analyzed by **B** genotyping, and **C** PCR using RNA extracted from bone marrow-derived macrophages. **D** Ulcer sizes (mm^2^) 1–7 days post-injury in control, SmoKO, control mice transplanted with bone marrow-derived from SmoKO mice (Control^SmoKO^), and SmoKO mice transplanted with bone marrow-derived from control mice (LysMCre/SmoKO^con^). Data are shown as the mean ± SEM. Representative H&E stains of **E** control, **F** SmoKO, **G** Control^SmoKO^, and **H** LysMCre/SmoKO^con^ mice.

A mouse model developed by our group expressing a myeloid cell-specific deletion of Smoothened (LysMcre/+;Smof/f, SmoKO) was generated using transgenic mice bearing loxP sites flanking exon 1 of the Smo gene (Smoflx/flx) and mice expressing a Cre recombinase transgene from the lysozyme locus (LysMCre)^14^ (Fig. 1A–C). Gastric ulcers were induced in control (LysMCre, Smof/f) or SmoKO mice using acetic acid according to our published protocols^7,8^ and mice were analyzed 1, 2, 3, 4, 5, and 7 days post-ulcer induction. Compared to control mice (black line, Fig. 1D, E), SmoKO mice (red line, Fig. 1D, F) had significantly larger ulcer sizes 7 days post-injury. To determine the role of gastric epithelial Shh as a macrophage chemoattractant during regeneration, bone marrow chimera experiments using SmoKO donor cells were performed. Compared to control mice, control chimera mice transplanted with SmoKO bone marrow donor cells exhibited a loss of ulcer repair (green line, Fig. 1D, G). Importantly, transplantation of control bone marrow donor cells to SmoKO mice rescued epithelial cell regeneration in these animals (blue line, Fig. 1D, H). These data demonstrate the importance of Hh signaling in the myeloid cell lineage during gastric epithelial cell regeneration and ulcer repair.

### SmoKO mice exhibit loss of macrophage infiltration to the site of injury

**Fig. 2:**
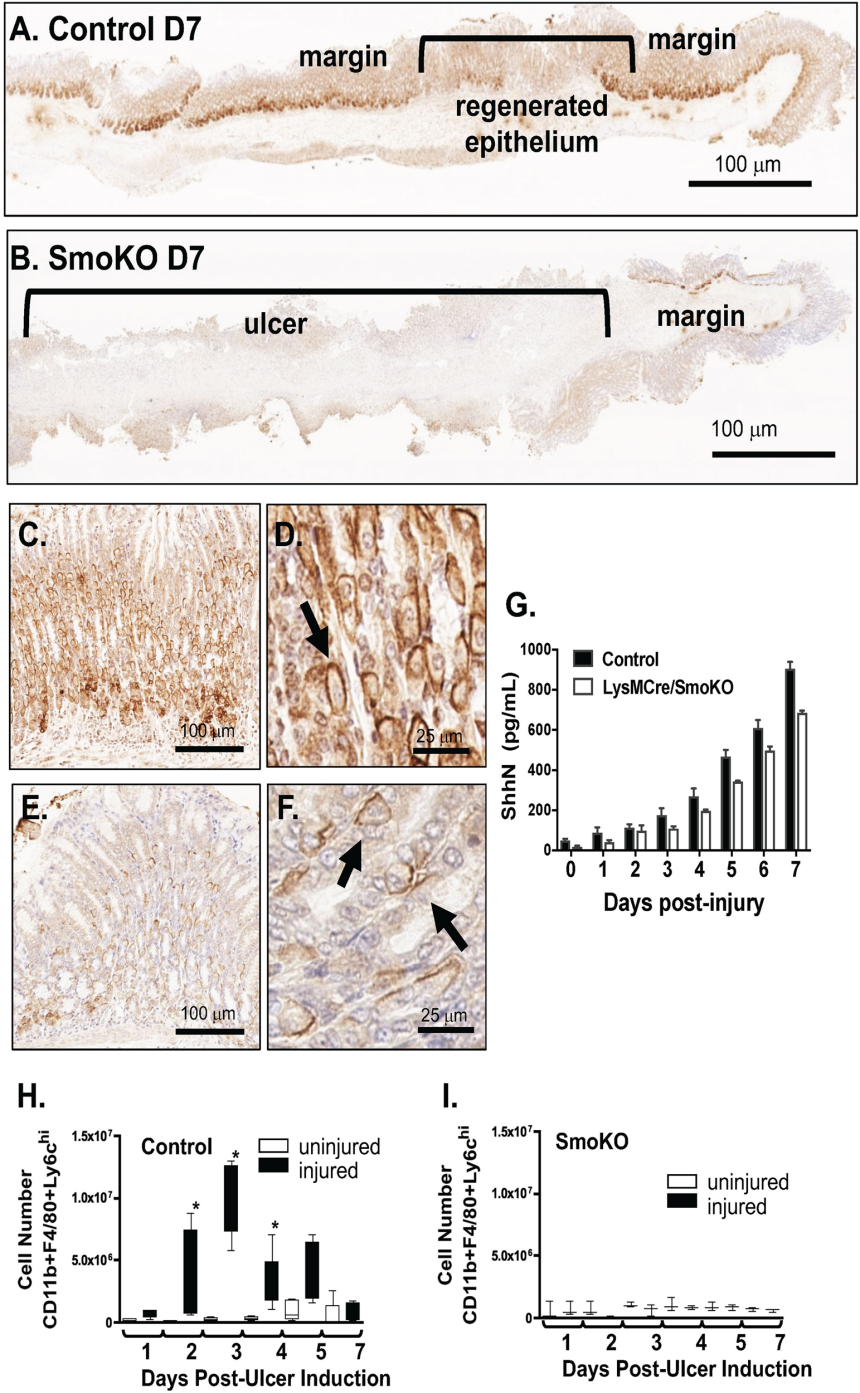
Expression of Shh and macrophage infiltration in control and LysMCre/SmoKO mice. Immunohistochemistry of Shh expression in **A** control and **B** LysMCre/SmoKO mouse stomachs. Shh expression at the ulcer margin of **C**, **D** control, and **E**, **F** LysMCre/SmoKO mouse stomachs. **G** Shh concentrations (pg/mL) measured in plasma collected from in control and LysMCre/SmoKO mice. Macrophage numbers (CD11b^+^F4/80^+^Ly6C^hi^) within the uninjured and injured gastric epithelium of **H** control and **I** LysMCre/SmoKO mice 1–7 post-ulcer induction. *P < 0.05 compared to day 1 post-injury, *n* = 6 mice per group. Data are shown as the mean ± SEM.

Within the stomach epithelium of both control (Fig. 2A) and SmoKO (Fig. 2B) mice, Shh protein expression was observed at the ulcer margins. Higher magnification of the ulcerated margins of control (Fig. 2C, D) and SmoKO (Fig. 2E, F) mice revealed the expression of Shh within parietal cells. Secretion of Shh in response to gastric injury was confirmed by a significant increase in circulating protein in blood collected from both control and SmoKO mice (Fig. 2G). Thus, the secretion of Shh from the epithelium is not affected within mice expressing a deletion in Smoothened within the myeloid cell lineage (SmoKO).

Stomachs were then enzymatically dissociated according to our previously published protocols^8,14^. Cells were labeled using antibodies against APC-conjugated F4/80, FITC-conjugated CD11b, PE-conjugated Ly6G, and PerCP-Cy5.5-conjugated Ly6C, and the number of gastric F480^+^/CD11b^+^/Ly6C^hi^/Ly6G^neg^ macrophages quantified by flow cytometric analysis according to our previously published protocols^14^. Control mice exhibited increased numbers of macrophages in the ulcerated tissue 2, 3 and, 4 days post-injury (Fig. 2H). Quantification of macrophages in the ulcerated tissue of SmoKO mice showed no significant increases in infiltrating cells compared to uninjured tissue (Fig. 2I). These data further support that macrophages are recruited to the site of gastric ulcer and that Hh signaling is needed for the recruitment of macrophages to the site of gastric injury.

### SmoKO mice exhibit loss of CD44v9 and IL-13 at the site of injury

**Fig. 3:**
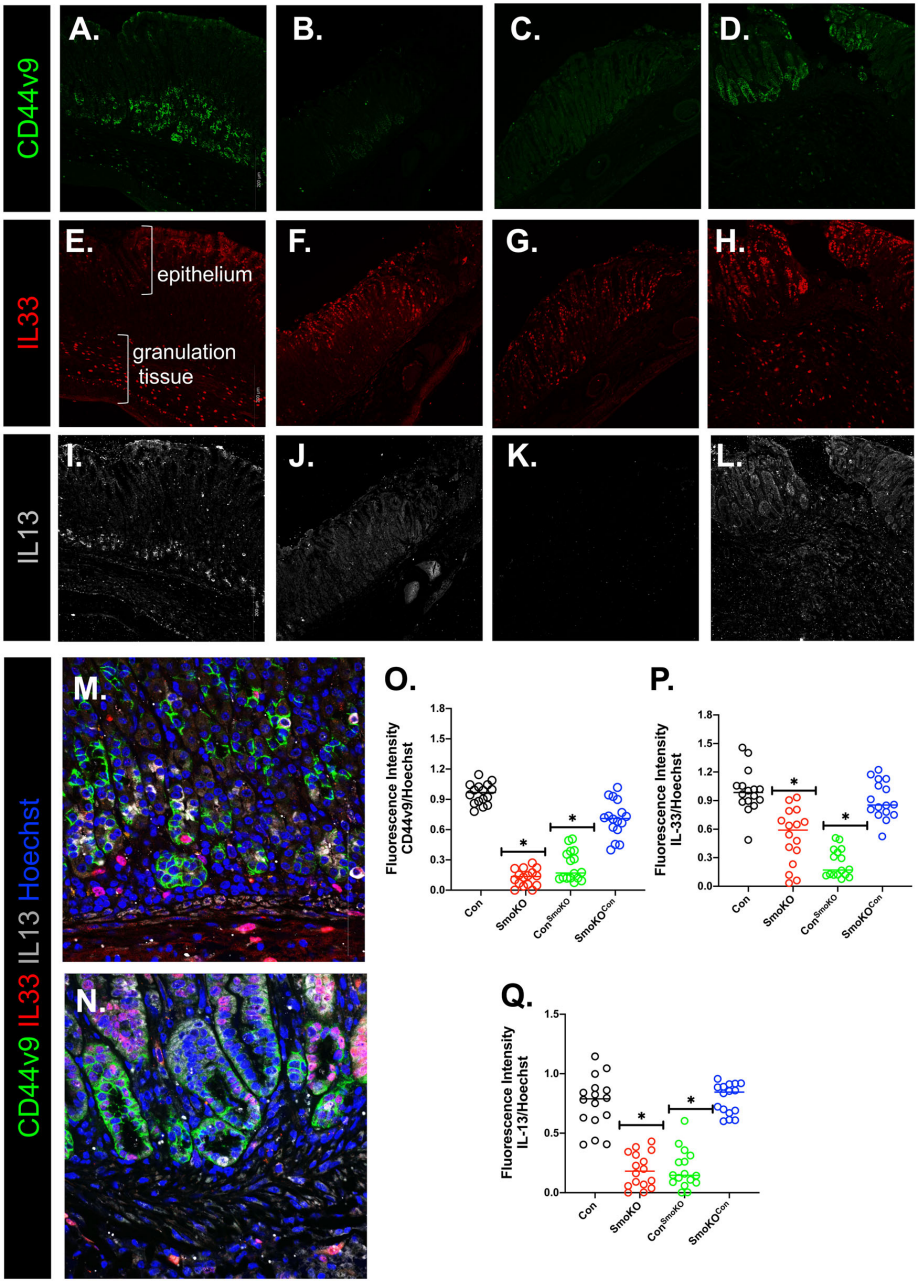
Expression of CD44v9, IL33, and IL13 within the stomachs of control and LysMCre/SmoKO mice. Representative immunofluorescence staining using antibodies specific for CD44v9 (green), IL33 (red), and IL13 (gray) in the stomachs of **A**, **E**, **I** control, **B, F**, **J** SmoKO, **C**, **G**, **K** Control^SmoKO^, and **D**, **H**, **L** LysMCre/SmoKO^con^ mice. Representative immunofluorescence staining using antibodies specific for CD44v9 (green), IL33 (red), and IL13 (gray) in the stomachs of **M** control and **N** LysMCre/SmoKO^con^ mice. Quantification of fluorescence intensity of **O** CD44v9, **P** IL33, and **Q** IL13 in mouse experimental groups. *P < 0.05 compared to control group, *n* = 6 mice per group. Data are shown as the mean ± SEM.

The induction of metaplasia has been shown to correlate with the infiltration of M2 macrophages and a cytokine signaling network of IL-33 and IL-13^15,16^. In particular, our group has demonstrated that the infiltration of macrophages to the gastric epithelium during bacterial infection and regeneration is dependent on Hh signaling^7,14^. In addition, CD44v9 marks a reparative cell lineage at the ulcer margin^16^. A loss of CD44v9 at the ulcer margin in SmoKO mice (Fig. 3B) compared to controls (Fig. 3A). Compared to control mice, control chimera mice transplanted with SmoKO bone marrow donor cells exhibited a loss of CD44v9 (Fig. 3C). Importantly, transplantation of control bone marrow donor cells to SmoKO mice rescued epithelial cell regeneration that correlated with the expression of CD44v9 (Fig. 3D). IL-33 was strongly expressed at the ulcer margin both within the epithelial cells and infiltrating cells in the granulation tissue of controls (Fig. 3E) and SmoKO mice transplanted with control bone marrow donor cells (Fig. 3H). However, IL-33 expression within the granulation tissue was lost in SmoKO mice (Fig. 3F) and control mice transplanted with SmoKO bone marrow donor cells (Fig. 3G). Loss of Smo signaling correlated with loss of infiltration of IL-13-expressing cells (Fig. 3J, K) compared to controls and SmoKO mice transplanted with control bone marrow donor cells (Fig. 3I, L, respectively). Higher magnification showed the expression of IL-33, IL-13, and CD44v9 in the control animals (Fig. 3M) and SmoKO mice (Fig. 3N) transplanted with control bone marrow donor cells. Quantification of the immunofluorescence intensity of all experimental mouse groups is shown in Fig. 3O–Q. These data demonstrate that Shh signaling drives the recruitment of macrophages and subsequently results in the production of IL-33 which then in part results in IL-13 secretion. IL-13 drives the epithelium to express CD44v9 and thus inducing the metaplasia regenerative response.

### Shh induces macrophage chemotaxis via a Smo-dependent mechanism

**Fig. 4:**
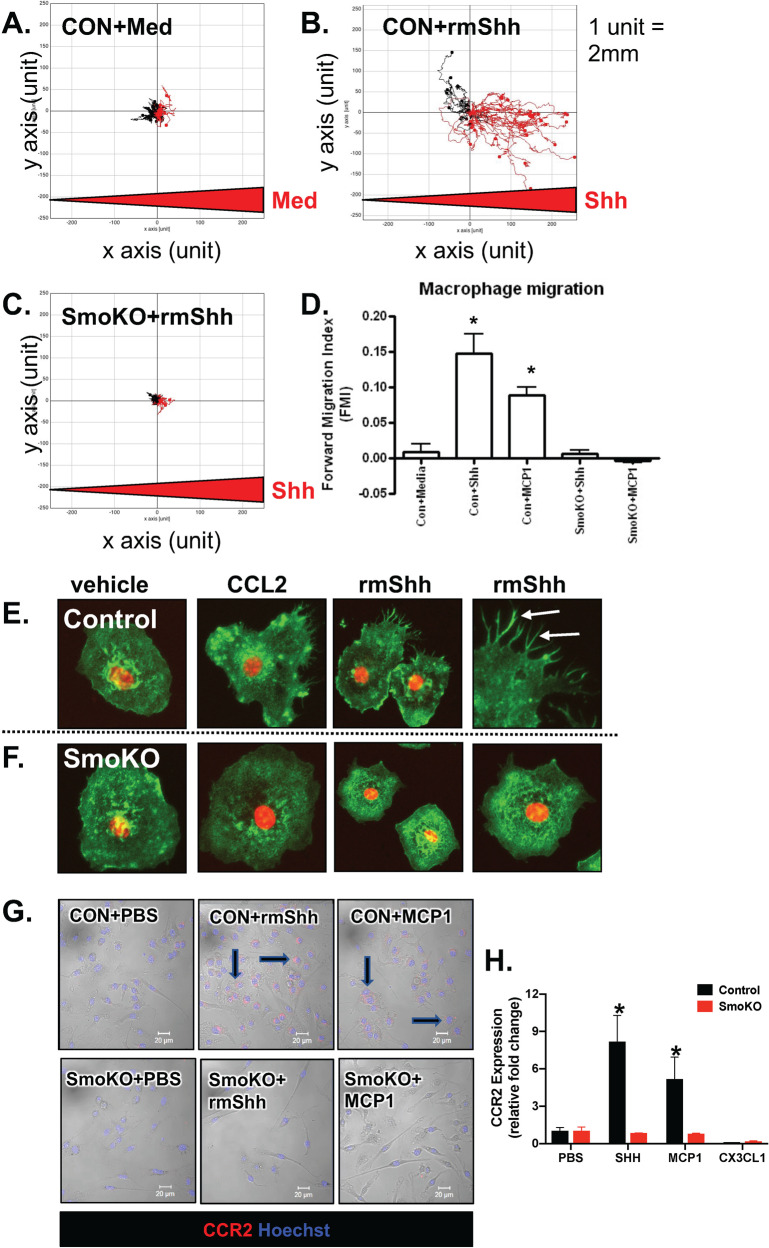
Macrophage migration and CCR2 expression. Migration plots of trajectories in response to **A** medium and **B**, **C** rmShh gradient using macrophages cultured from the bone marrow of control and SmoKO mice. **D** Macrophage migration is represented as forward migration index (FMI). Representative immunofluorescence stains of actin (green) and nuclei (red) of bone marrow-derived macrophages cultured from **E** control and **F** SmoKO mice treated with vehicle, CCL2, or rmShh. Arrows indicate filopodia. **G** Representative immunofluorescence stain of CCR2 (red) and nuclei (Hoechst, blue) of bone marrow-derived macrophages cultured from control or SmoKO mice and treated with PBS, rmShh, or MCP. **H** Quantitative RT-PCR of CCR2 expression using RNA collected from control or SmoKO bone marrow-derived mouse macrophages and treated with PBS, Shh, MCP1, or CXC3L1. *P < 0.05 compared to PBS vehicle control, *n* = 6 individual cultures. Data are shown as the mean ± SEM.

We have shown that mice lacking parietal cell-expressed Shh have a deficient immune response to *H. pylori* infection and that the Hh signaling transducer, Smo, is required for macrophage migration to the infected stomach^14^. To identify the mechanism by which Shh elicits macrophage chemotaxis, we used an in vitro migration assay using cultured bone marrow-derived macrophages (BM-MΦs) isolated from both control and SmoKO mice. Control BM-MΦs faced with no chemokine exhibited non-directional movement (Fig. 4A, D), whereas rmShh induced chemotaxis (Fig. 4B, D). Control BM-MΦs migrated toward the chemokine MCP1 gradient as expected (Fig. 4D). We found that BM-MΦs that lack Smo expression (SmoKO) were unable to migrate toward either MCP1 or rmShh and exhibited limited non-directional movement (Fig. 4C, D). These findings demonstrate chemotaxis in response to Shh ligand-dependent upon the Smo receptor for this effect. Interestingly it was also observed that chemotaxis towards the established macrophage chemokine, MCP1, was also impaired in BM-MΦs lacking the Smo receptor (Fig. 4D). This unexpected finding indicated that Smo may play a role in transducing chemotactic signals not only from Shh ligand but also other chemotactic signals. In coordination with stress fiber and retraction fiber formation, these projections along the leading edge propel macrophages toward the chemokine. Compared to macrophages derived from control mice (Fig. 4E), treatment of SmoKO macrophages with CCL2 or Shh did not alter cell morphology and cells retained morphology lacking F-actin filopodia (Fig. 4F). These findings demonstrate a requirement for Smo in cellular responses to chemokine ligand, CCL2, and Shh, and show that this crucial step in cellular locomotion is deficient in cells lacking Hh signaling via Smoothened.

### Shh induces CCR2 expression in MΦs via Smo signaling

The chemokine receptor, CCR2, is expressed by macrophages and is known to play a crucial role in the response of monocyte/macrophage chemotaxis to infected tissue^17,18^. Figure 4G, H demonstrate an increase in CCR2 expression in response to rmShh and positive control MCP in macrophages isolated and cultured from the bone marrow of control animals. In contrast to the control experiments, CCR2 expression in macrophages isolated and cultured from SmoKO mice did not increase in response to either rmShh or MCP1 (Fig. 4H). Immunofluorescence data was confirmed by measuring significantly increased CCR2 expression in response to rmShh and MCP1 in cultured control macrophages (Fig. 4G). These data suggest that Hh signals, similar to CCL2/MCP1, elicit downstream signaling that enhances macrophage migration.

**Fig. 5:**
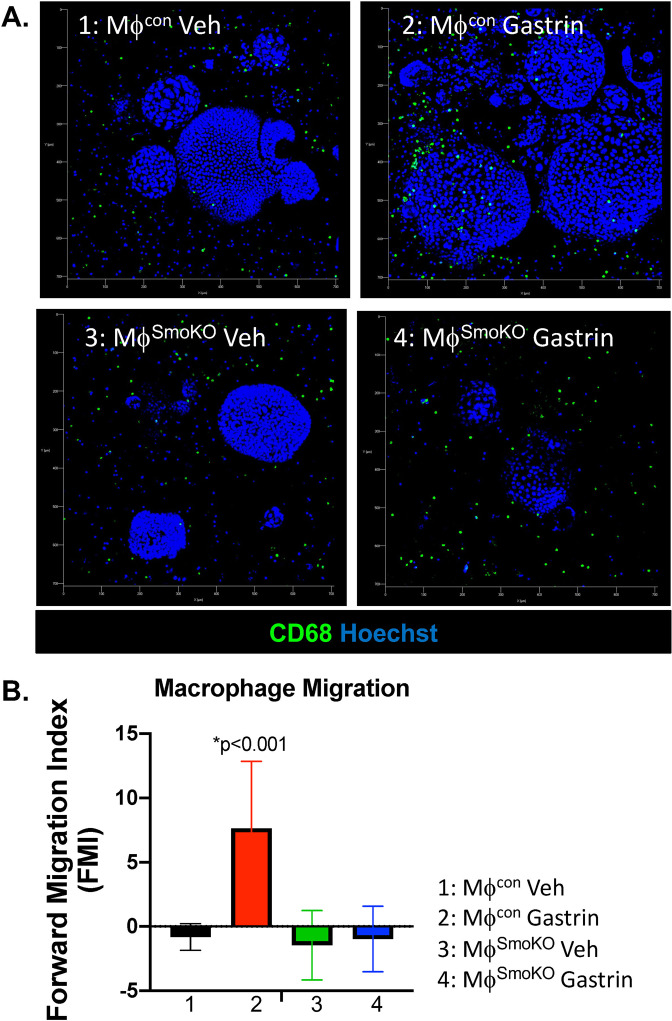
Macrophage migration in an organoid/macrophage co-culture model. **A** Immunofluorescence imaging demonstrating migration of CD68+ macrophages cultured from control (MΦ^con^) and SmoKO (MΦ^SmoKO^) mice towards mouse gastric organoids. Co-cultures were treated with vehicle or gastrin. **B** Macrophage migration within the co-cultures was quantified by forward migration index (FMI). *P < 0.001 compared to vehicle, *n* = 6 individual co-culture experiments. Data are shown as the mean ± SEM.

Macrophage chemotaxis in response to Hh signaling was then investigated in a gastric-derived organoid/macrophage co-culture generated using macrophages isolated from control (Con) or SmoKO mice (Fig. 5). We used gastrin, known to stimulate Shh secretion from gastric parietal cells to induce macrophage chemotaxis. Immunofluorescence staining showed migration of macrophages towards the gastric organoids stimulated with gastrin compared to the vehicle control (Fig. 5A, B). Macrophages with loss of Smo expression did not exhibit migration in response to gastrin (Fig. 5A, B). These data further supported the essential role of Hh signaling in macrophage chemotaxis toward the gastric epithelium.

### Shh signaling induces macrophage chemotaxis in human-derived organoid/MΦ co-cultures

**Fig. 6:**
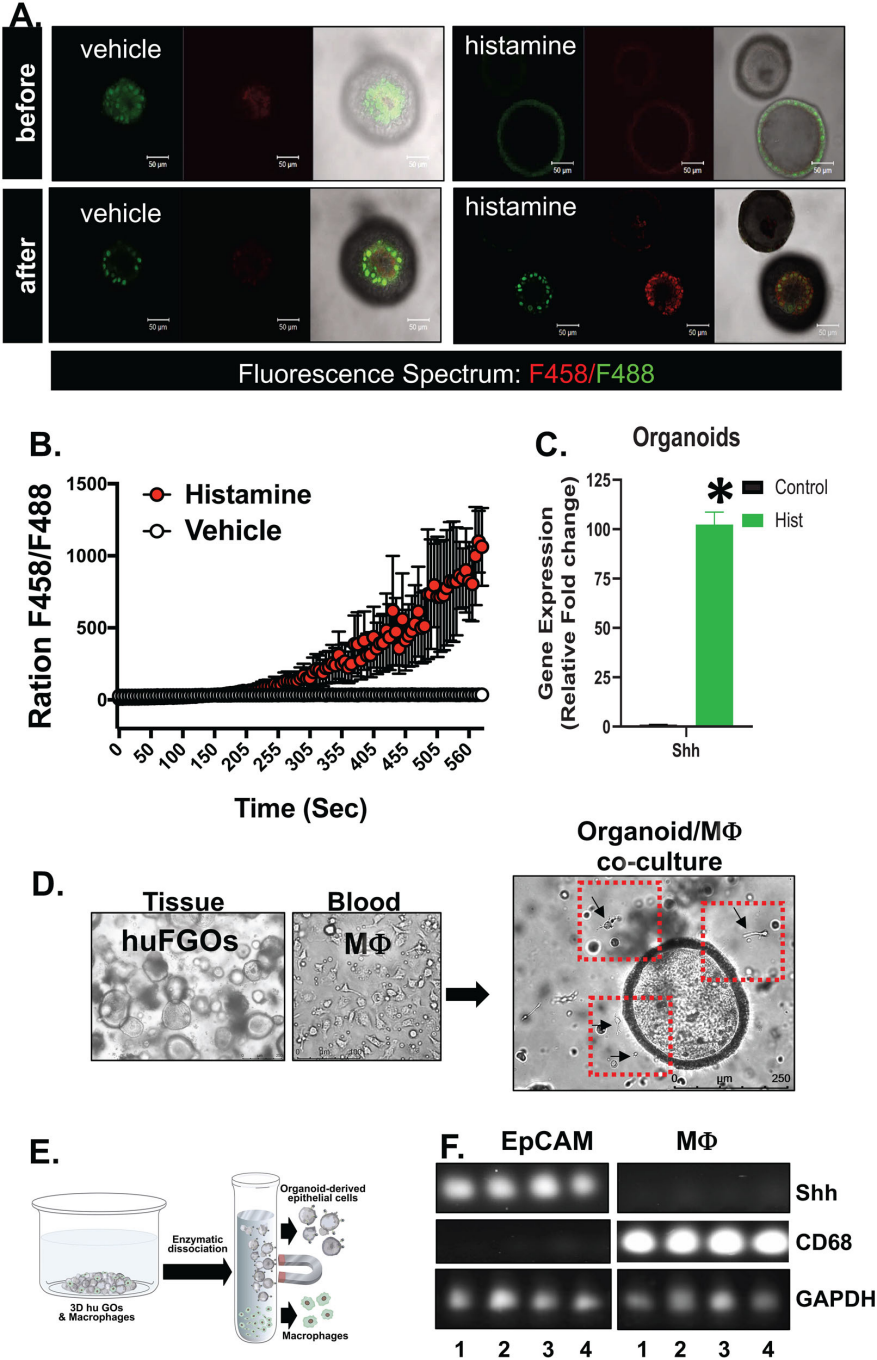
Human gastric organoid/macrophage co-cultures. **A** Acridine orange assay using gastric organoids treated with either vehicle or histamine. **B** Shift in F458 (red)/F488 (green) fluorescence ratio was measured in the organoid cultures treated with vehicle or histamine. **C** Quantitative RT-PCR measuring changes in Shh expression in the gastric organoids treated with vehicle (control) or histamine (Hist). *P < 0.05 compared to control, *n* = 4 cultures generated from four individual patients. **D** Bright-field micrographs showing tissue-derived human fundic gastric organoids (huFGOs), PBMC-derived macrophages, and representative co-culture. **E** Magnetic separation was used to isolate epithelial and immune cells from treated human gastric organoid/macrophage co-cultures. **F** RT-PCR demonstrating expression of Shh in EpCAM positive and CD68 in macrophage fractions. Data are shown as the mean ± SEM.

Parietal cell-expressed Shh is processed from a 45 kDa to a 19 kDa bioactive protein via a mechanism that is acid- and protease-dependent^19–21^. Acridine Orange is a green fluorescence (F488) dye at a neutral pH and shifts in the fluorescent spectrum to red (F458) when it accumulates in acidic organelles, such as the parietal cell secretory canaliculus^22^. In response to histamine, Acridine Orange accumulated in cell vesicles, within human-derived gastric organoids, as shown by an increase in the shift in red fluorescence and increase in the ratio of F458 (red)/F488 (green) (Fig. 6A, B). This increase in the ratio of F458 (red)/F488 (green) was not observed in vehicle-treated organoids (Fig. 6A, B). The increase in acid secretion from histamine-treated gastric organoids correlated with a significant increase in Shh expression (Fig. 6C).

To study the chemotaxis of macrophages toward the human gastric epithelium in response to Shh secretion, we established a gastric organoid/macrophage co-culture (Fig. 6D). Supplementary Video 1 shows the migration of macrophages toward the gastric epithelium in response to histamine-stimulated Shh secretion. Magnetic separation was then used to isolate the organoid-derived epithelial cells and macrophages from each condition of the co-culture (Fig. 6E). PCR of Shh and CD68 showed a clear separation of these cell populations after separation (Fig. 6F).

**Fig. 7:**
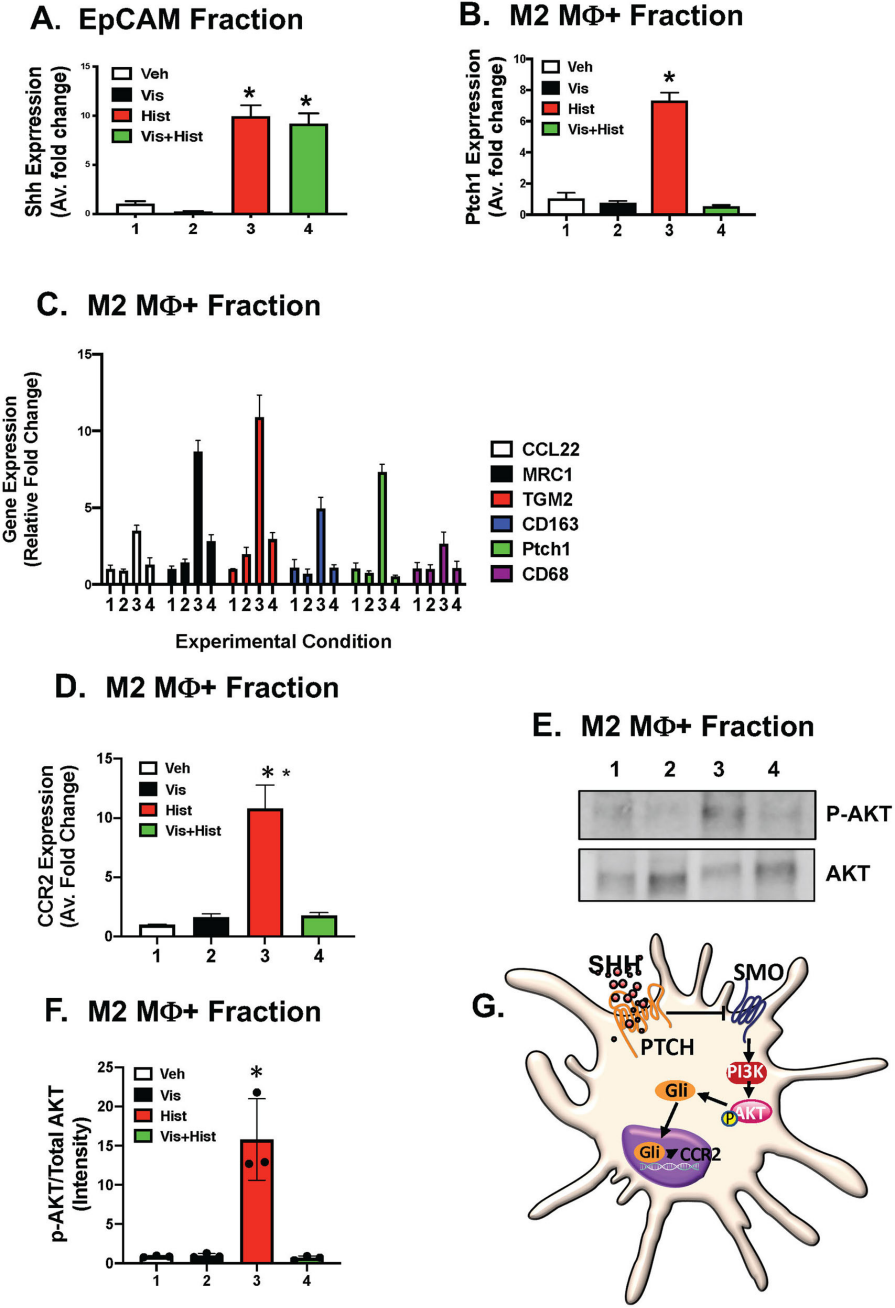
Expression of Shh, Ptch1, CCR2, and M1/M2 macrophage markers in organoid/macrophage co-cultures. **A** Expression of Shh in EpCAM fractions isolated from vehicle (Veh, 1), vismodegib (Vis, 2), Histamine (Hist, 3), or Vis+Hist (4) treated co-cultures. **B** Expression of Ptch1 in MΦ fractions isolated from Veh, Vis, Hist, or Vis+Hist treated co-cultures. **C** Expression of M1 and M2 markers in MΦ fractions isolated from Veh, Vis, Hist, or Vis+Hist treated co-cultures. **D** Expression of CCR2 in MΦ fractions isolated from Veh, Vis, Hist, or Vis+Hist treated co-cultures. **E** Protein expression of AKT and pAKT in MΦ fractions isolated from Veh, Vis, Hist, or Vis+Hist treated co-cultures. Quantification of immunoblots in **E** is shown in (**F**)**. G** Schematic diagram showing Shh-induced macrophage migration that is Smo-dependent is mediated by AKT signaling. *P < 0.05 compared to vehicle. Data are shown as the mean ± SEM.

Quantitative RT-PCR showed significant induction in Shh expression within the organoid epithelium in response to histamine with or without treatment with the smoothened inhibitor, Vis (Fig. 7A). Migration of macrophages toward the gastric epithelium in response to histamine-induced Shh secretion, correlated with significantly increase Gli target gene Ptch1 specifically in the macrophage fraction of the co-culture (Fig. 7B). Pretreatment of the co-cultures with Vis resulted in blockade of Ptch1 expression and macrophage chemotaxis in response to histamine-induced Shh expression (Fig. 7B). Induction of Hh signaling correlated with a significant increase in CCR2 expression specifically within the M2 macrophage fraction (Fig. 7C, D).

The PI3K/Akt pathway plays a fundamental role in the survival, proliferation, and migration of macrophages (reviewed in ref. ^23^), and published studies have shown that PI3-kinase and Akt signaling are indeed essential for Hh signaling^24^. Western blot analysis of the macrophage fraction isolated from the co-cultures revealed a significant increase in P-AKT in response to histamine-induced Shh expression (Fig. 7E, F). Smo inhibitor Vis blocked P-AKT that was induced by histamine-induced Shh expression (Fig. 7E, F). Collectively, these data suggest that Shh secreted from the gastric epithelium, induces macrophage chemotaxis via a Smo-dependent pathway that is mediated by Akt signaling (Fig. 7G).

### Inhibition of Smo signaling via chemical or genetic knockdown leads to decreased CCR2 expression

**Fig. 8:**
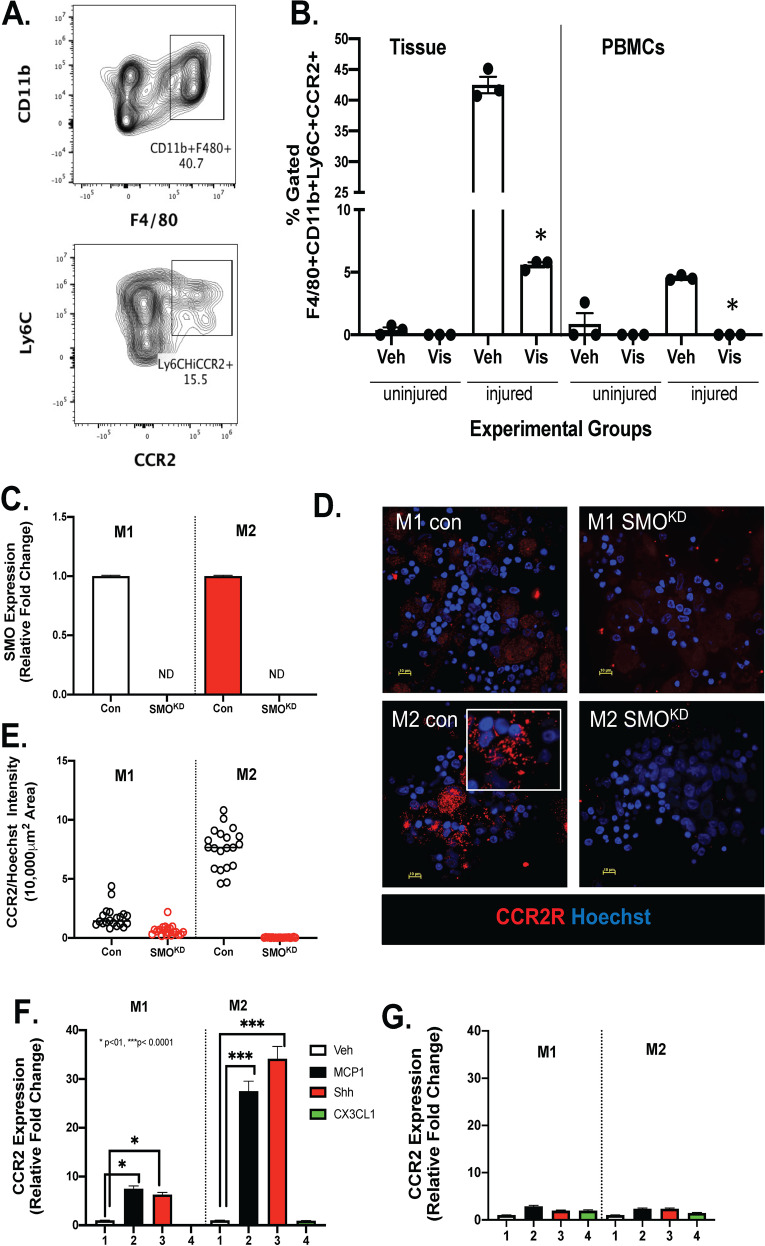
Changes in CCR2 in mouse and human monocytes in response to Smo chemical inhibition or gene knockdown. **A** Flow cytometric contour plots demonstrating the gating strategy for quantification of CD11b+F4/80+Ly6C^Hi^CCR2+ macrophages. **B** Quantification of flow cytometry analysis for the expression of CD11b+F4/80+Ly6C^Hi^CCR2+ macrophages within tissues and PBMCs collected in response to acetic acid-induced injury. Uninjured controls were subjected to surgery and stomachs exposed to PBS. **C** Quantitative RT-PCR documenting knockdown of Smoothened (SMO^KD^) in human-derived monocyte cultures. **D** Immunofluorescence of M1 or M2 macrophages in control and SMO^KD^ cultures for CCR2 expression (red). **E** Fluorescence intensity was measured using images captured in (**D**). Quantitative RT-PCR using RNA collected from (**F**) control and (**G**) SMO^KD^ cultures polarized to either M1 or M2 macrophages and treated with vehicle (Veh), MCP-1, Shh, or CX3CL1. *P < 0.05 compared to control or vehicle groups, *n* = 3 individual biological replicates. Data are shown as the mean ± SEM.

To investigate the effect of Shh signaling on the expression of CCR2, we took both in vivo and in vitro approaches (Fig. 8). After the acetic acid-induced injury, mice were treated with either vehicle control or Hh/Smoothened inhibitor vismodegib. Flow cytometry was used to gate for CD11b+/F4/80+/Ly6^hi^ CCR2 expressing macrophages (Fig. 8A). Flow cytometric analysis revealed that compared to the uninjured stomach tissue, there was significant infiltration of CCR2-expressing macrophages. CCR2 expression was significantly inhibited in the stomach tissue within the injured site in response to vismodegib (Fig. 8B). Peripheral blood mononuclear cells (PBMCs) collected from the acetic acid-induced injured group revealed a significant increase in CD11b+/F4/80+/Ly6^hi^ CCR2-expressing macrophages, and importantly this expression was blocked using vismodegib (Fig. 8B). These data supported the notion that Smo alters the differentiation of monocytes at the level of the bone marrow and blood. To test our hypothesis, we used human-derived monocytes to knockdown smoothened (SMO^KD^) (Fig. 8C). Monocytes were then polarized to either an M1 or M2 phenotype. While CCR2 expression was significantly lower in the control M1 macrophages compared to the M2 cultures, SMO^KD^ cultures showed significantly lower expression levels of the receptor as quantified by immunofluorescence (Fig. 8D, E). Quantitative RT-PCR showed that both MCP-1 and Shh significantly induced CCR2 in both M1 and M2 polarized macrophage cultures (Fig. 8F), and this response was absent in SMO^KD^-treated cultures (Fig. 8G). Collectively, these data demonstrate that Smo plays an important role in the differentiation and expression of CCR2 at the monocyte level.

### Shh signaling mediates IL-33 secretion and subsequent IL-13 release

**Fig. 9:**
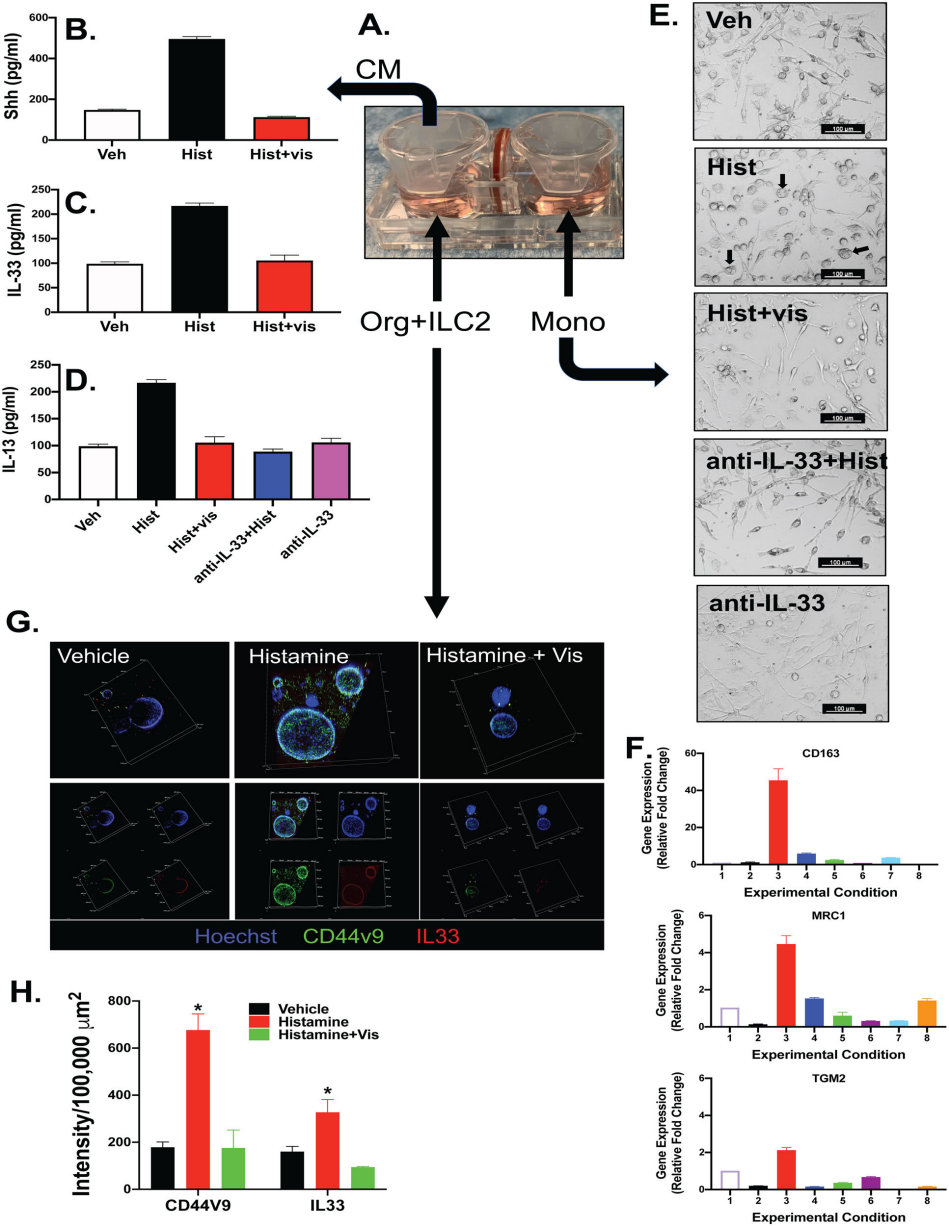
Organoid/ILC2/monocyte Interactive Co-Culture Plates. **A** Interactive Co-Culture Plates were used to culture human gastric-derived organoids and ILC2s in the left chamber and monocytes in the right chamber. Conditioned medium collected from the organoid/ILC2 co-culture was used to measure changes in **B** Shh, **C** IL-33, and **D** IL-13 secretion. **E** Bright-field micrographs of monocytes after treatment with the vehicle, histamine, histamine + vismodegib, anti-IL-33 immuneutralization antibody + Histamine, or anti-IL-33 alone. **F** Quantitative RT-PCR was used to measure changes in M1/M2 markers in the monocyte cultures. Expression of M2 markers in monocyte chamber isolated from Veh (1), Vis (2), Hist (3) or Vis+Hist (4), anti-IL33 (5), anti-IL33 + Hist (6), anti-IL13 (7), and anti-IL13 + Hist (8), treated co-cultures. **G** Immunofluorescence staining for expression of CD44v9 (green), IL-33 (red), and Hoechst (blue) in organoid/ILC2 co-cultures treated with vehicle, histamine or histamine + Vismodegib. **H** Fluorescence intensity for CD44v9 and IL-33 was quantified in co-cultures. *P < 0.05 compared to vehicle, *n* = 3 co-cultures prepared from three individual patients. Data are shown as the mean ± SEM.

The Interactive Co-Culture Plate was used to study the role of Hh signaling in macrophage polarization and IL-33 and IL-13 release (Fig. 9A). Human gastric-derived organoids were cultured together with isolated ILC2 cells, and monocytes were seeded on the opposite chamber (Fig. 9A). Collection of conditioned media from the chamber with organoid/ILC2 co-cultures revealed that histamine-induced significant secretion of Shh that was blocked by Smo inhibitor vismodegib (Fig. 9B). Similarly, IL-33 (Fig. 9C) and IL-13 Fig. 9D) secretion was induced by histamine, a response that was also blocked by vismodegib. Immunoneutralization of the cultures using an anti-IL-33 antibody resulted in the significant inhibition of IL-13 secretion induced by histamine (Fig. 9D). Immunofluorescence showed a significant increase in CD44v9 and IL-33 expression within the organoid gastric epithelium in response to histamine (Fig. 9G, H). Expression of histamine-induced CD44v9 and IL-33 was blocked with vismodegib pretreatment (Fig. 9G, H). Analysis of monocytes seeded in the opposite chamber of the Interactive Co-Culture Plate showed the induction of monocytes to an M2 macrophage polarization with the expression of M2 marker CD163, MRC1, and TGM2, and this response was blocked by Smo inhibitor vismodegib and anti-IL-33 and anti-IL-13 immunoneutralization (Fig. 9E, F). Collectively, these data show that histamine-induced Shh signaling mediates IL-33 expression within the gastric epithelium and ILC2s with subsequent release of IL-13 that drives M2 macrophage polarization.

## Discussion

The current study demonstrates that Shh plays a fundamental role in the chemotaxis of macrophages to the gastric epithelium in response to injury. These data build on our work that has placed the Hh signaling pathway amongst the key factors that regulate the regeneration of the gastric epithelium during repair^7,8^. Published studies have clearly shown that Shh regulates epithelial cell differentiation^2,5,25^. Shh, which is secreted from parietal cells^19,26^, regulates the differentiation of cell lineages and controls gastric physiological function^27^. The development of a mouse model expressing a parietal cell-specific deletion of Shh (HKCre/Shh^KO^) has allowed us to assay changes in gastric epithelial cell differentiation and function^27^. Our laboratory has documented that Hh ligand Shh acts as a macrophage chemoattractant during *H. pylori* infection^7,8,14^. These findings are advanced by using a mouse model expressing a deletion of Smo within the myeloid cell lineage demonstrating that Shh-induced macrophage migration to the site of injury is mediated by Smo signaling within the macrophages. Typically, inflammatory cues within the regional microenvironment prime the macrophage into a reparative phenotype. Inflammatory monocytes are recruited in response to cytokine cues and undergo differentiation into two distinct subsets of macrophages that are categorized as either classically activated (M1) or alternatively activated (M2)^10^. M2 macrophages represent various phenotypes that are further subdivided into M2a, M2b, and M2c^28^. T_H_2 cytokines induce the polarization of macrophages to an M2 phenotype^29^. Therefore, using the LysMCre/SmoKO mouse model and human-derived organoid/macrophage co-cultures, we find that Shh plays a fundamental role as a macrophage chemoattractant during the repair.

Loss of Shh signaling in the myeloid cell lineage observed in the LysMcre/+;Smof/f (SmoKO) mice correlated with not only a loss of macrophage recruitment to the injured stomach but also disruption of epithelial cell regeneration. Induction of spasmolytic polypeptide/TFF2-expressing metaplasia (SPEM) is critical to driving regeneration of the gastric epithelium^16,30^. Cluster-of-differentiation gene 44 variant isoform 9 (CD44v9) emerges during regeneration of the gastric epithelium in response to injury and is known to drive SPEM glands. In fact, CD44v9-expressing SPEM marks a regenerative lineage^16^. Macrophages are key regulators for repair and regeneration^13^. Earlier studies show that Shh secreted from the gastric parietal cells may act as an endocrine factor that induces macrophage migration to the stomach in response to infection and injury^7,8^. In support of a macrophage chemoattractant role for Shh, SmoKO mice exhibited decreased macrophage infiltration that coincided with the failure to repair and regenerate the gastric epithelium in response to injury. The lack of this response correlated with the loss of the CD44v9-expressing SPEM regenerative lineage at the sites of injury in mice that lacked recruitment of macrophages.

We observed a significant induction in IL-33 expression within the gastric epithelium and granulation tissue in response to injury, and this response was lost within the SmoKO mice. IL-33 is an alarmin for the rapid induction of IL-13-driven immunity^31,32^. It is known that IL-13 and IL-4 mediate alternative M2 macrophage activation through a common receptor IL-4Ra^29^. Mice that lack the expression of parietal cell-derived Shh exhibit diminished growth factor and cytokine responses following injury and thus suggests that Shh may be upstream of factors that promote tissue repair including IL-33^7^. IL-33 was expressed within the epithelium and infiltrating cells in the granulation tissue. IL-13 produced by group 2 innate lymphoid cells (ILC2s), is downstream of IL-33 and is known to regulate gastric metaplasia^33^ and intestinal stem cell renewal^34^. Blocking IL-33 in the organoid/ILC2/monocytes interactive co-cultures using a neutralization antibody, resulted in the loss of IL-13 secretion and polarization of the monocytes to an M2 macrophage phenotype. These findings are supported in the context of parietal cell atrophy, whereby IL-33 is necessary to promote a Th2 inflammatory response leading to M2 macrophage polarization that subsequently drives the induction of SPEM^35^. IL-13 is downstream of IL-33 as a critical regulator of the development of SPEM^33,35,36^. We observe a similar response in the context of regeneration in response to acute injury.

Analysis of macrophages isolated from the organoid/immune cell co-cultures showed that Shh-induced macrophage chemotaxis is Smo-dependent and mediated by Akt signaling. The PI3K/Akt pathway plays a fundamental role in the survival, proliferation, and migration of macrophages (reviewed in ref. ^23^), and published studies have shown that PI3-kinase and Akt signaling are indeed essential for Hh signaling^24^. In addition, actin rearrangement within macrophages was also Smo-dependent. Macrophage migration requires a coordinated set of cytoskeletal rearrangements directed by the Rac/Rho family of GTPases and their involvement in cellular polarization activities^37^. Furthermore, Shh plays an important role in tissue patterning, in part mediated by its effects on cytoskeletal rearrangements, and has been linked to the formation of actin stress fibers^38^. The cycling of intracellular GTPases likely plays a role in the velocity of the cells with increasing amounts of Rac-GTP being linked with increased cell velocity^39^. Bone marrow-derived macrophages from SmoKO mice had an FMI of ~0 towards rmShh indicating non-directional movement. Furthermore, SmoKO mouse-generated bone marrow macrophages did not migrate toward the MCP/CCL2 chemokine gradient and also had an FMI of ~0. Interestingly, SmoKO macrophages had a baseline velocity ~40% slower than control macrophages and these findings suggest that a deficiency in macrophage mobility may result from a lack of Smo in addition to a deficiency in directed migration. Taken together with the knowledge that Smo can regulate the actin cytoskeleton, these results demonstrate that in addition to Smo being a necessary receptor for the Mø chemotactic response to Shh and CCL2 chemokine, this ligand may also be involved in processes that regulate baseline cell movement.

**Fig. 10:**
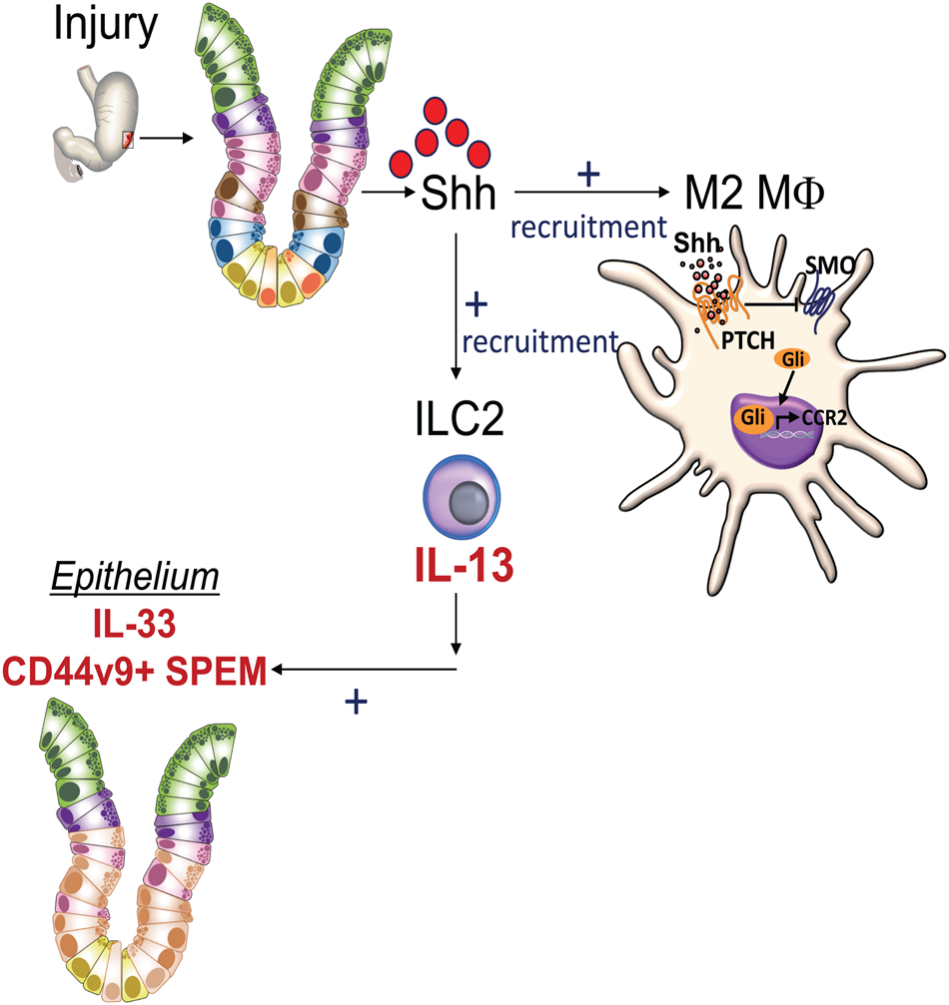
Schematic diagram showing the role of Shh as a macrophage chemoattractant during regeneration of the gastric epithelium. Shh, secreted from the gastric parietal cells in response to acute injury to the stomach induced macrophage chemotaxis and subsequently CD44v9-expressing SPEM. Shh acts as a macrophage chemoattractant via a smoothened-dependent mechanism. IL-33 is released from the injured epithelium, and macrophages drive the polarization of macrophages to an M2 phenotype. IL-13, released from recruited ILC2 cells, subsequently regulates the development of CD44v9-expressing SPEM, which is crucial for the regeneration of the gastric epithelium.

In summary, our investigation demonstrates that Shh, secreted from the gastric parietal cells in response to acute injury to the stomach^7^, is fundamental to regeneration of the epithelium. Regeneration is regulated by Shh-induced macrophage chemotaxis and subsequent CD44v9-expressing reparative SPEM. Shh acts as a macrophage chemoattractant via a smoothened-dependent mechanism within the myeloid cell lineage that results in the induction of CCR2 expression with the M2 macrophages. IL-33 released from the injured epithelium, and also macrophages, may drive the Th2 cytokine response necessary for the polarization of macrophages to an M2 phenotype. IL-33 may also be secreted by M2 macrophages to induce IL-13 secretion by ILC2 cells, and subsequently activate M2 macrophages. IL-13, which is downstream of IL-33 and released from recruited ILC2 cells, subsequently regulates the development of CD44v9-expressing SPEM, which is crucial for the regeneration of the gastric epithelium (Fig. 10)^16,30^.

## Methods

### Mice

All mouse studies were approved by the University of Cincinnati Institutional Animal Care and Use Committee (IACUC) that maintains an American Association of Assessment and Accreditation of Laboratory Animal Care (AAALAC) facility (Protocol number 19-571). The LysMCre/SmoKO mice were generated by crossing the LysMCre (The Jackson Laboratory, B6.129P2-Lyz2tm1(cre)Ifo/J, stock number 004781) with the Smoflx/flx (The Jackson Laboratory, Smotm2Amc/J, stock number 004526 | Smo^c^) mice. The LysMCre knock-in allele has a nuclear-localized Cre recombinase inserted into the first coding ATG of the lysozyme 2 gene (Lyz2). When crossed with Smoflx/flx mice, that have loxP sites flanking exon 1 of the Smo gene, Cre-mediated recombination results in deletion of the targeted gene in the myeloid cell lineage, including monocytes, mature macrophages, and granulocytes^14^. LysMCre mice were genotyped using primers 5′-CCC AGA AAT GCC AGA TTA CG-3′ and 5′-CTT GGG CTG CCA GAA TTT CTC-3′ and for wild-type LysM using primer pair 5′-TTA CAG TCG GCC AGG CTG AC-3′ and 5′-CTT GGG CTG CCA GAA TTT CTC-3′. Smoflx/flx mice are genotyped using primers 5′-CTT GGG TGG AGA GGC TAT TC-3′ and 5′-AGG TGA GAT GAC AGG AGA TC-3′ and for Smoothened-wild type using primer pair 5′-CCA CTG CGA GCC TTT GCG CTA C-3′ and 5′-CCC ATC ACC TCC GCG TCG CA-3′. PCR thermocycler conditions for LysMCre and LysM gene are 94 °C (1 cycle, 3 min), 35 cycles of 94 °C (30 s), 62 °C (1 min), and 72 °C (1 min), and then one final cycle of 72 °C for 2 min. PCR conditions for Smoflx/flx are as: 94 °C (1 cycle, 3 min), 35 cycles of 94 °C (30 s), 55 °C (30 s), and 72 °C (30 s), and then one final cycle of 72 °C for 2 min. Conditions for Smoothened-wild type are as: 94 °C (1 cycle, 3 min), 35 cycles of 94 °C (30 s), 65 °C (30 s), and 72 °C (30 s), and then one final cycle of 72 °C for 2 min.

### Acetic acid-induced injury model

Gastric ulcers were induced in control (LysMCre, Smoflx/flx) or LysMcre/+;Smof/f (SmoKO) mice using acetic acid according to published protocols^7,8^. Briefly, after mice were anesthetized using isoflurane, a ventral midline abdominal laparotomy was performed to exteriorize the stomach. Glacial acetic acid (~25 µL of 100%) or sterile phosphate-buffered saline was applied to the serosal surface for 25 s using a 2 mm diameter capillary tube. In a separate series of experiments, mice were treated with either vehicle (0.01% DMSO/PBS) or vismodegib (5 mg/kg, oral gavage for 3 days after induction of ulcer). Mice were analyzed 1, 2, 3, 4, 5, and 7 days post-ulcer induction.

### Bone marrow chimera experiments

To determine the role of gastric epithelial Shh as a macrophage chemoattractant during epithelial repair, bone marrow chimera experiments were performed using LysMCre/SmoKO mice and donor cells. Two experimental groups were used that included: (1) control recipient (LysMCre or Smoflx/flx) mice transplanted with LysMcre/+;Smof/f donor cells (control^SmoKO^) and (2) LysMcre/+;Smof/f recipient mice transplanted with control donor cells (SmoKO^cont^). Bone marrow chimera experiments were performed according to a previously published protocol^14^. Briefly, recipient mice were irradiated with 900 rads from a cesium 137-gamma cell irradiator. Up to 8 h after irradiation, donor bone marrow was injected via a single tail vein injection. Donor cells were collected from the bone marrow of euthanized mice. Mice were given antibiotics in drinking water 5 days prior to irradiation and then for up to 14 days after irradiation. After 4 weeks of recovery, acetic acid ulcers were induced as described above, and mice were analyzed 1, 2, 3, 4, 5, and 7 days post-ulcer induction.

### Mouse bone marrow-derived macrophage cultures

Mouse macrophages were derived from bone marrow cells collected from either control or LysMcre/+;Smof/f mice. The bone marrow was collected from the femur and tibia, filtered through a 40 μm filter, centrifuged at 1200 rpm for 5 min at 4 °C. Cells were resuspended in macrophage media (DMEM high glucose 1×, 10% fetal calf serum, 1% P/S) supplemented with 40 ng/mL mouse MCSF (Peprotech, AF-315-02). 50% of media were replaced with fresh media on day 3 and supplemented with 40 ng/mL mouse MCSF. On day 6, MCSF was withdrawn from the culture. On day 7, both control and LysMcre/+;Smof/f macrophages were treated with either DPBS, recombinant SHH (R&D Systems, 464-SH, 1 μg/mL), MCP1 (R&D Systems, 479-JE/CF, 100 ng/mL), or CX3CL1 (R&D Systems, 472-FF/CF, 100 ng/mL). Macrophages were harvested after 48 h for the analysis of qRT PCR or western blot.

### Human-derived gastric organoid/macrophage co-cultures

Human fundus/corpus tissue was collected from sleeve gastrectomies (IRB protocol number: 2015-5537, University of Cincinnati and 2014-0427, Cincinnati Children’s Hospital Medical Center; IRB protocol number: 1912208231R001, University of Arizona Human Subjects Protection Program), and gastric organoids generated from isolated glands according to previously published protocols^30,40^. Briefly, epithelial tissue was separated from the muscle layer, cut into small fragments, and washed in Dulbecco’s phosphate-buffered saline without Ca^2+^/Mg^2+^ (DPBS). Tissue fragments were placed 1 mg/mL Collagenase 1A (Millipore Sigma) from *Clostridium histolyticum* and Bovine Serum Albumin (2 mg/mL) for 30 min at 37 ^o^C. Isolated gastric glands were then suspended in 50 μL Matrigel (Thermo Fisher), and cultured in human gastric organoid media (DMEM/F12 (Thermo Fisher), HEPES (10 mM), 1× l-glutamine (Thermo Fisher), 1× Pen/Strep, 1× N2 (Thermo Fisher), 1× B27 (Thermo Fisher), N-acetylcysteine (1 mM; Sigma), Nicotinamide (10 mM; Sigma), EGF (50 ng/mL; Peprotech), Noggin (100 ng/mL; Peprotech), 10% R-spondin conditioned media, 50% Wnt conditioned media, FGF10 (200 ng/mL; Peprotech), Gastrin (1 nM, Tocris), Y-27632 (10 μM; Sigma). Organoids were harvested after 4–7 days of growth.

### Interactive co-culture plates

The sterile and disposable interactive co-culture plates (ICCP, Advantigen Biosciences, SKU: 2501-02AB) were used to set up the co-culture with organoids, ILC2 cells, and the macrophages. Two separate wells, A and B were connected side-by-side via an O-ring and filter system which allows two-cell populations to share growth media and secretion factors without cross-contamination. Human type 2 innate immune cells (ILC2) were isolated from PBMCs using EasySep^TM^ Human ILC2 Enrichment kit (Stem Cell Technologies, 17972) following manufacturers’ protocol and were cultured in α-MEM media, supplemented with 50 ng/mL of IL-33, IL-25, and 20 IU/mL IL-2 for 10 days. In order to set up Organoid/ILC2/macrophage co-cultures, hFGO organoids were harvested in cold DPBS and centrifuged at 400×*g* for 5 min. ILC2 cells were collected in DPBS and centrifuged at 400×*g* for 5 min. ILC2 and hFGO were combined and re-plated together in Matrigel^TM^ dome in well A and supplemented with complete organoid growth media. Macrophages were also harvested at the same time in 0.25% Trypsin/EDTA, centrifuged at 1200 × rpm for 5 min, re-seeded in well B, and cultured in macrophage base media (DMEM high glucose 1×, 10% fetal calf serum, 1% P/S) without MCSF. 24 h later the organoid/ILC2 co-culture was pretreated with vismodegib for 1 h. histamine was added to the co-culture either alone or in combination with vismodegib for 48 h. Cultures from both wells were clearly visible and imaged under a microscope. Conditioned media were collected from well A and organoid/ILC2 co-culture were fixed in 4% paraformaldehyde. The macrophages were harvested from well B in TRIzol reagent (Molecular Research Center, TR118) and RNA was extracted following manufacturers’ instruction and analyzed by qRT-PCR.

### Macrophage culture

Human macrophages were generated from PBMCs, extracted from the whole blood of the autologous patient using LymphoprepTM density gradient medium (STEMCELL Technologies # 07851). Isolated lymphocytes were plated in macrophage media (DMEM high glucose 1X, 10% fetal calf serum, 1% P/S) supplemented with 100 ng/mL human MCSF (Peprotech, AF-300-25). 50% media were replaced with fresh media on day 3 and supplemented with 100 ng/mL human MCSF. On day 6, MCSF was withdrawn from the culture. Macrophages were polarized to either M1 or M2 phenotypes on day 7 by treating with 2 ng/mL IFN-g (ThermoFisher Scientific) and 100 ng/mL LPS (Millipore SIGMA) or 10 ng/mL IL-4 (ThermoFisher Scientific). The polarized macrophages were used for co-culture after 24 h.

Organoid/macrophage co-cultures were generated using hFGO organoids harvested in cold DPBS and centrifuged at 400×*g* for 5 min. Macrophages were also harvested at the same time in 0.25% Trypsin/EDTA and centrifuged at 1200×rpm for 5 min. Macrophages and hFGO were combined and re-plated together in Matrigel^TM^ dome and supplemented with complete organoid growth media. 24 h later the organoid/macrophage co-culture was pretreated with vismodegib (Selleckchem, S1082) to the desired wells for 1 h. histamine (Millipore SIGMA, H7125) was added to the co-culture either alone or in combination with vismodegib. 48 h later, organoids were harvested separately from the macrophages using a biotinylated EpCAM antibody (Invitrogen, 13-5791-82) bound to magnetic beads and CELLectin Biotin Binder kit (Invitrogen, 11533D). EpCAM-positive organoids and EpCAM-negative macrophages were analyzed by qRT PCR and western blot.

### Smoothened knockdown in human monocytes

Human peripheral blood mononuclear cells (PBMCs) were cultured in monocyte media (DMEM high glucose, 10% fetal calf serum, 1% P/S) for 48 h in 12-well cell culture plates. Monocytes were transfected with SMO gene-specific predesigned gRNA (SIGMA, HSPD0000039249) 5 μg of SMO gRNA were complexed with 5 μg of Cas9 protein (SIGMA) and incubated on ice for 30 min. gRNA/Cas9 complex was then incubated with 8 mg/mL Polybrene (Sigma), and Lipofectamine LTX plasmid transfection reagent (Thermo Fisher Scientific) following manufacturer’s protocol. The mixture was then added dropwise to the monocytes. After 24 h incubation at 37 °C, an additional 1 mL of complete monocyte media was added. Most of the culture medium was aspirated and replaced by fresh monocyte media supplemented with 100 ng/mL human MCSF (Peprotech) in order to differentiate to macrophages. Cells were grown for 6 more days, and media was replenished every other day. On day 7 MCSF was withdrawn from the culture. Macrophages were then polarized to either M1 or M2 phenotypes on day 7 by treating with either 2 ng/mL IFN-g and 100 ng/mL LPS (for M1) or 10 ng/mL IL-4 (for M2). The polarized macrophages were ready to use for downstream experiments after 48 h of further culture.

### Immunohistochemistry and immunofluorescence

Stomach sections spanning both the fundic and antral regions were collected from mice and fixed for 16 h in 4% paraformaldehyde, paraffin-embedded, and sectioned at 5 µM. Prior to immunostaining, slides were deparaffinized and antigen retrieval was performed by boiling slides in a 1:100 dilution of antigen unmasking solution (Vector Laboratories, H-3300) for 10 min followed by a 20 min cooling at room temperature. Sections were incubated for 30 min in 0.3% hydrogen peroxide/methanol, blocked for 1 h with horse serum, and immunostained with 1:200 goat anti-Shhh (R&D systems, AF464), 1:200 mouse anti F480 (BioRad, MCA497) overnight at 4 °C. Slides were then incubated with biotinylated anti-mouse or anti-goat affinity-purified anti IgG secondary antibodies for 30 min and subsequently incubated with ABC reagent (Vectastain ABC Kit; Vector Laboratories) for 30 min. The color was developed with 3,3′-diaminobenzidine (DAB) using the DAB Substrate Kit (Vector Laboratories, Burlingame, CA) and then counterstained with hematoxylin (Fisher Scientific Company, Kalamazoo, MI). Slides were then dehydrated and mounted using Permount and images viewed and captured under light microscopy (Leica Biosystems, Aperio AT2).

Whole-mount staining of gastric organoids derived from fresh tissue was performed as previously described^40^. Briefly, organoids were fixed in 3.7% formaldehyde for 15 min at room temperature. Organoids were permeabilized with 0.5% Triton X-100 for 20 min at room temperature. Organoids were incubated with primary antibody overnight and washed in PBS containing 0.01% Triton-X 100. Secondary antibody incubation was also performed overnight in gastric organoids, and subsequently immunostained for cell nuclei using 10 μg/mL Hoechst. The following primary antibodies and dilutions were used: 1:100 mouse anti-CCR2 (Novus Biologicals, NBP1-48338), 1:500 mouse anti-CD68 (Novus Biologicals, NBP2-37265) slides, and whole-mount organoids were imaged on a Zeiss LSM710 LIVE Duo Confocal Microscope.

### Acridine orange assay

Acridine orange (Millipore SIGMA, A9231) was added to the organoid culture, and the assay was measured in response to either vehicle or histamine (6.67 mM, Sigma Aldrich) stimulation. Images were analyzed using the Zeiss LSM710 Microscope and background corrected 550–620/620–700 nm ratio values were converted to fold change corresponding to pH change using Prism Graph Pad software.

### Flow cytometry

Cells were collected from uninured and injured tissue digest, centrifuge at 400×*g* for 5 min, and were resuspended in PBS with 5% bovine serum albumin. Cells were then incubated with CD16/CD32 FcBlock (BD Biosciences, 553141) for 5 min at 4 °C. Cells were labeled using a 1:100 concentration of antibodies against APC-conjugated F4/80 (Thermo Fisher Scientific, MF48005), FITC-conjugated CD11b (Thermo Fisher Scientific, RM2801), PE-conjugated Ly6G (BD Biosciences, 551461), and PerCP-Cy5.5-conjugated Ly6C (eBiosciences, 45-5932) for 20 min at room temperature in dark and then fixed with fixative medium A (Thermo Fisher Scientific, GAS004) for 15 min at room temperature. Unstained and single-stained cells controls were used as compensation controls. Cells were then washed and resuspended in 500 μL of DPBS containing 0.5% fetal bovine serum and analyzed using FACS Calibur flow cytometer (BD Biosciences). The number of gastric F480^+^/CD11b^+^/Ly6C^hi^/Ly6G^neg^ macrophages were quantified using FlowJo software according to our previously published protocol^14^.

### Macrophage migration assay and analysis

Time-lapse imaging of macrophage migration was performed using tissue culture treated μ-Slide Chemotaxis 2D slides (ibidi, #80306). Macrophages were seeded into the chamber in serum-free DMEM at a density of 1 × 10^6^ cells/mL. Cells were allowed to attach before media, CCL2 (100 ng/mL in DMEM) or recombinant mouse Shh (1 μg/mL in DMEM), was loaded into one side of the chemotaxis chamber. Cells were monitored for 16 h using a ZEISS LSM710 confocal microscope at 37 °C and 5% CO_2_ under transmitted light, with images captured every 3 min. Cell migration and velocity were analyzed using ImageJ (National Institutes of Health, USA) and the Manual Tracking plugin. Each frame was analyzed and the weighted center of macrophages was identified. Velocity was measured by calculating average distance over time and forward migration index (FMI) was calculated by measuring average distance on the *x*-axis over average total distance. A positive FMI would indicate migration towards the chemoattractant whereas a negative FMI indicated migration away from the chemoattractant.

### Western blot

Cell lysates were mixed (1:1 v/v) with Laemmli loading buffer plus β-mercaptoethanol (Bio-Rad Laboratories, CA) before western blots were performed. Samples were then loaded into 4–20% Tris–glycine gradient gels (Invitrogen) and ran at 80 V for 2.5 h at room temperature and transferred to nitrocellulose membranes (Whatman Protran, 0.45 µM) at 105 V for 1 h 15 min at 4 °C. Membranes were then blocked at room temperature using KPL Detector Block Solution (Kirkegaard & Perry Laboratories, Inc.) for 1 h and subsequently incubated at 4 °C for 16 h with a 1:1000 dilution of anti-GAPDH (Thermo Fisher Scientific), 1:1000 anti-AKT (Cell Signaling), 1:1000 phospho AKT (Cell Signaling), followed by 1 h incubation with a 1:1000 dilution anti-mouse, anti-rabbit, or anti-rat Alexa Fluor 680 (Invitrogen) at room temperature. Blots were imaged using a scanning densitometer along with analysis software (Odyssey Infrared Imaging Software System) and the ratio of individual antibody/GAPDH was calculated using ImageJ.

### Shh ELISA

Blood (300–700 μL) was collected by cardiac puncture from both acetic acid injured control and LysMCre/SmoKO mouse at 1, 2, 3, 4, 5, and 7 days post-ulcer induction. Blood was centrifuged at 3000 rpm for 15 min, and circulating Shh concentrations were measured using a mouse ELISA specific for the N-terminus of biologically active processed Shh following the manufacturer’s protocol (RayBioTech, ELH-ShhN-1). Concentrations were calculated from standard curves using recombinant proteins and expressed in pg/mL.

### Quantitative RT-PCR

RNA was isolated using TRIzol Reagent (Molecular Research Center, TR118) according to the manufacturer’s instructions. The High Capacity cDNA Reverse Transcription Kit (Applied Biosystems) was used for cDNA synthesis of RNA following the manufacturer’s protocol. For each sample, 100 ng of RNA was reverse transcribed to yield ~2 μg total cDNA that was then used for the real-time PCR. Pre-designed Real-Time PCR assays were purchased for the following genes (Thermo Fisher, Applied Biosystems): Mouse (Mm)-specific GAPDH (Mm99999915_g1), CCR2 (Mm99999051_gH), SMO (Mm01162710_m1) or human (Hs)-specific PTCH1 (Hs00181117_m1), SHH (Hs00179843_m1), CD68 (Hs02836816_g1), SOCS1 (Hs00705164_s1), CCL22 (Hs01574247_m1), MRC1 (Hs00267207_m1), TGM2 (Hs01096681_m1), CD163 (Hs00174705_m1), GAPDH (Hs02786624_g1). PCR amplifications were performed in a total volume of 20 μL, containing 20× TaqMan Expression Assay primers, 2× TaqMan Universal Master Mix (Applied Biosystems, TaqMan® Gene Expression Systems), and cDNA template. Each PCR amplification was performed in duplicate wells in a StepOne™ Real-Time PCR System (Applied Biosystems), using the following conditions: 50 °C 2 min, 95 °C 10 min, 95 °C 15 s (denature) and 60 °C 1 min (anneal/extend) for 40 cycles. Fold change was calculated as: (Ct–Ct high) = ntarget, 2ntarget/2nHPRT = fold change, where Ct = threshold cycle. The results were expressed as average fold change in gene expression relative to control with GAPDH used as an internal control according to Livak and Schmittgen^41^.

### Statistics

The significance of the results was tested by one-way ANOVA or Student’s *t*-test using commercially available software (GraphPad Prism Software, San Diego, CA). A *P* value < 0.05 was considered as the level of significance.

### Reporting summary

Further information on research design is available in the Nature Research Reporting Summary linked to this article.

## Supplementary information

Supplementary Information

Supplementary Movie 1

Reporting Summary

## Data Availability

The datasets generated during and/or analyzed during the current study are available at: Chakrabarti, Jayati; Dua-Awereh, Martha; Schumacher, Michael; C. Engevik, Amy; Zavros, Yana (2021): Zavros-NPJ Regen Med. University of Arizona Research Data Repository: 10.25422/azu.data.16798171.
