# Supplementary material for: Sonic Hedgehog acts as a macrophage chemoattractant during regeneration of the gastric epithelium
Source: NPJ Regen Med. 2022 Jan 12;7:3. doi: 10.1038/s41536-021-00196-2 (PMC8755719; doi:10.1038/s41536-021-00196-2)

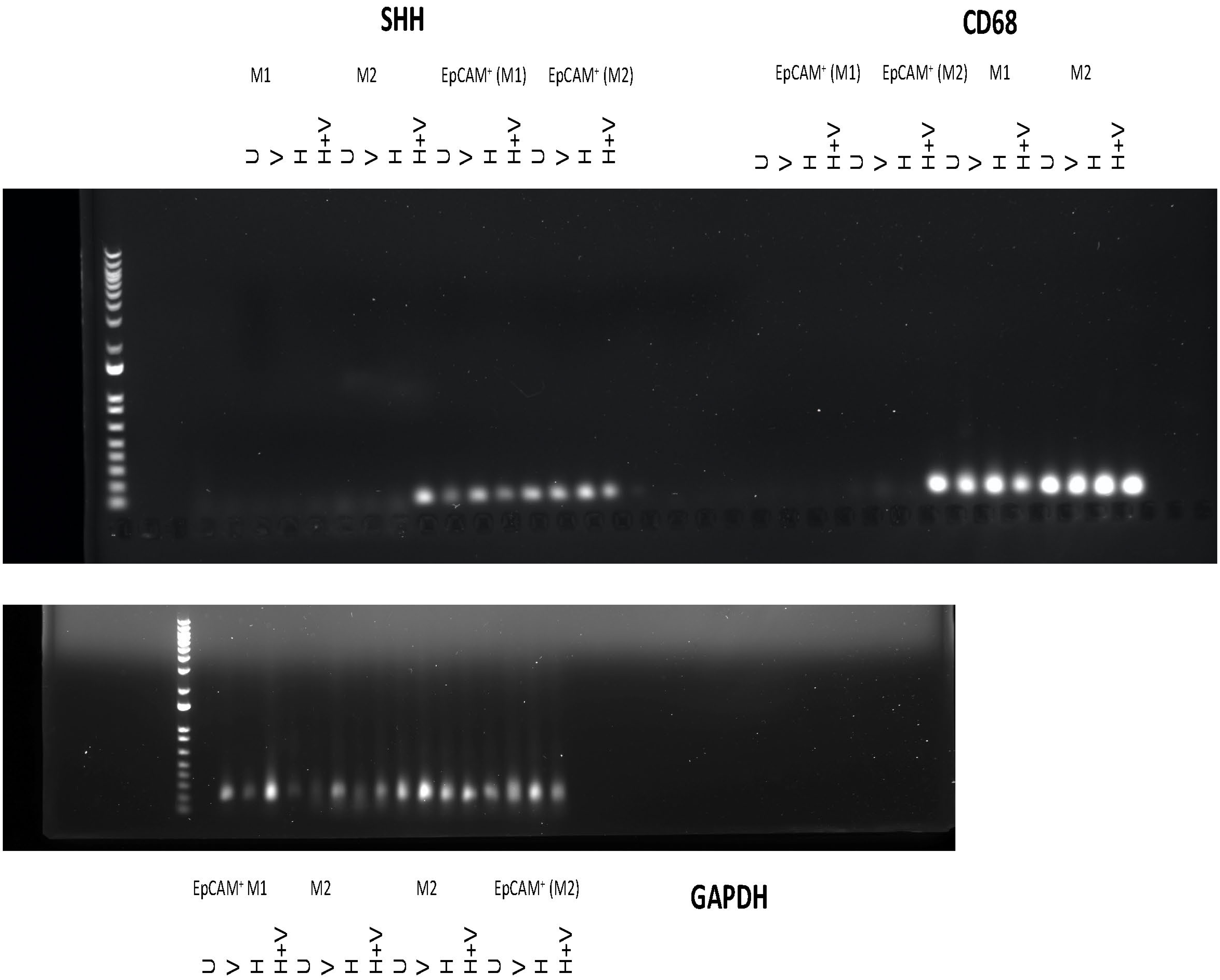
SHH
CD68
M1
M2
EpCAM+ (M1)
EpCAM+ (M2)
EpCAM+ (M1)
EpCAM+ (M2)
M1
M2
EpCAM+ M1
M2
M2
EpCAM+ (M2)
GAPDH

P-AKT (approximately 60kDa)

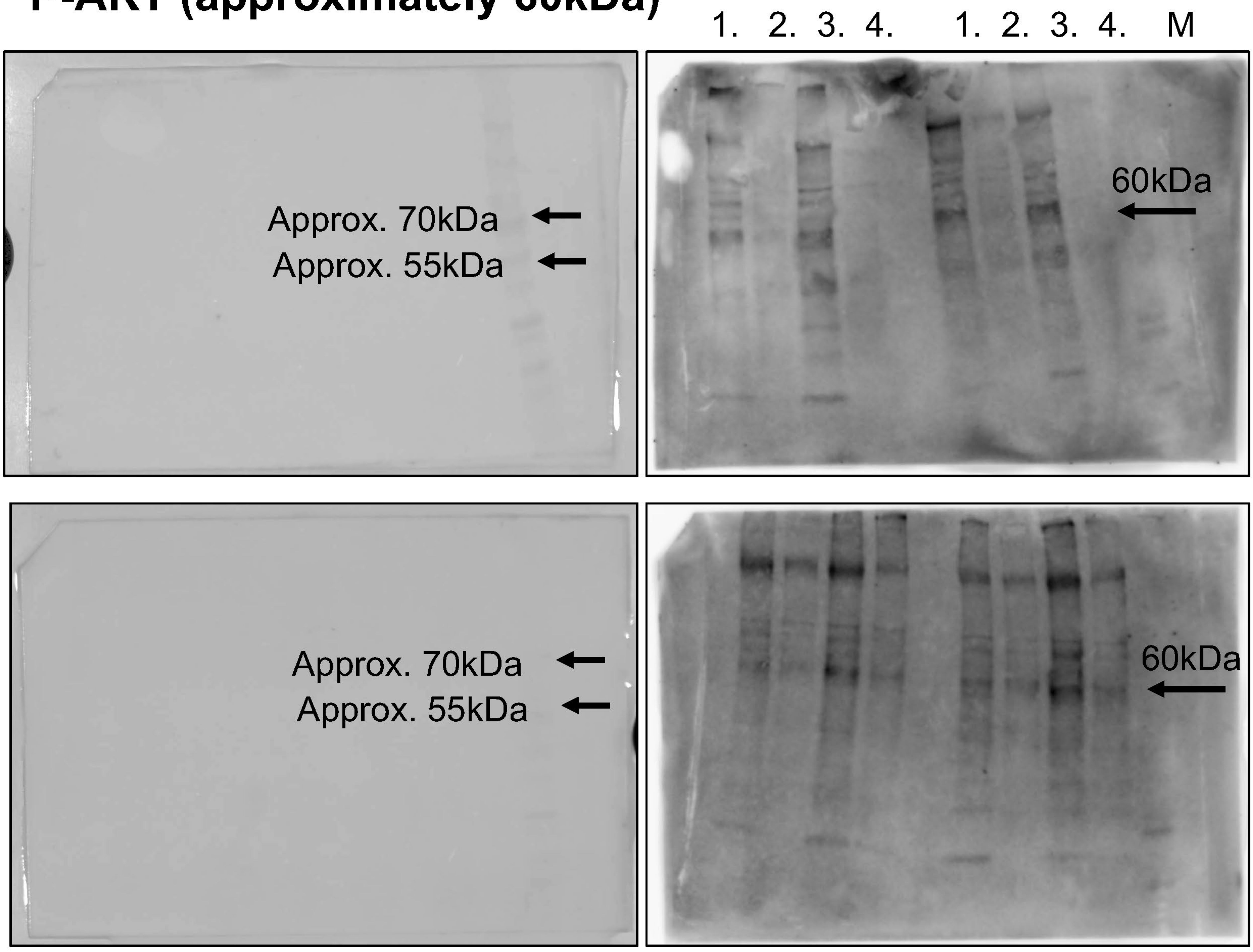

AKT (approximately 60kDa)

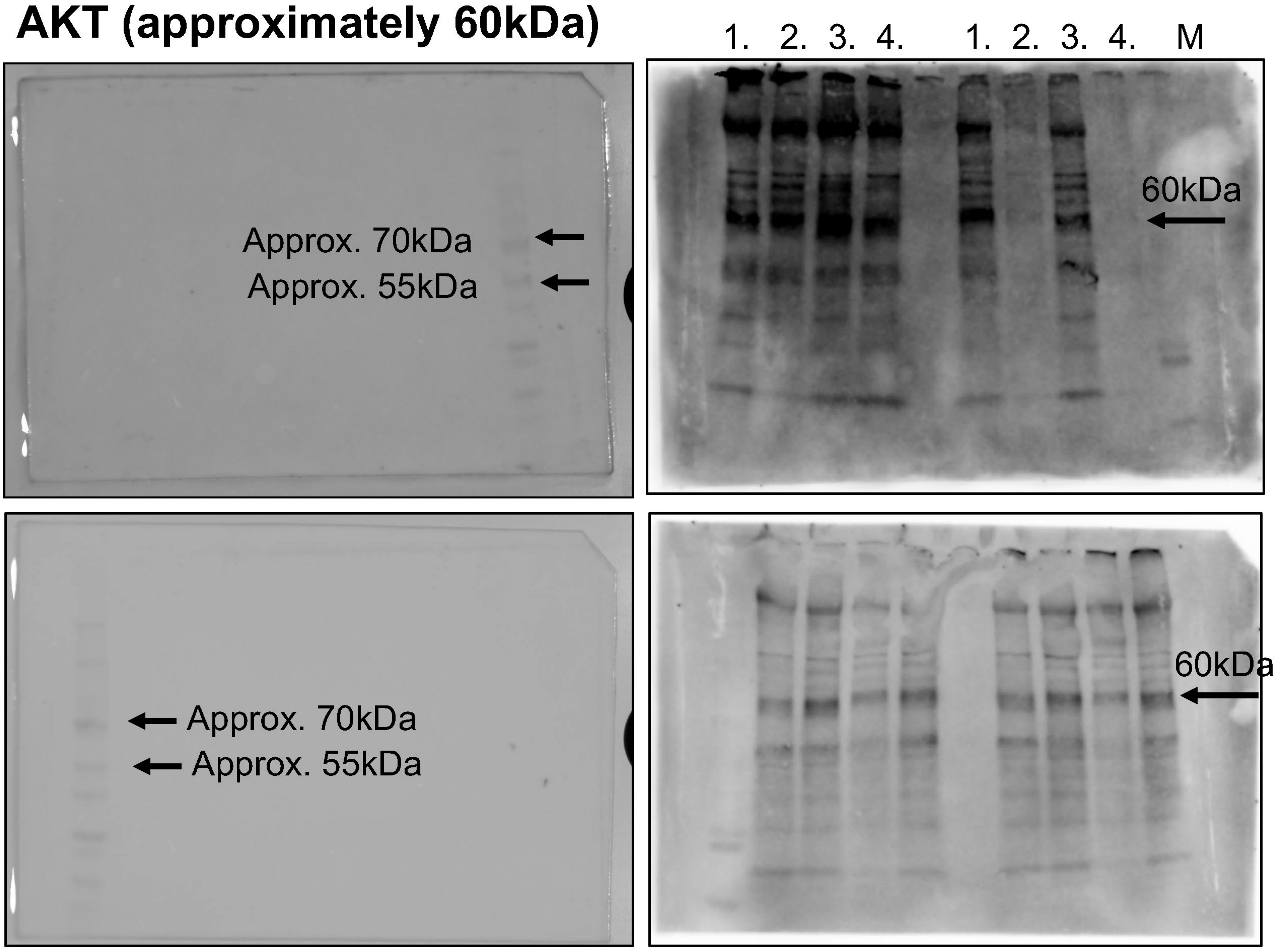

Supplement: Supplementary file 1 — Supplementary Information [file 41536_2021_196_MOESM1_ESM.pdf]
