# Supplementary material for: Sonic Hedgehog acts as a macrophage chemoattractant during regeneration of the gastric epithelium
Source: NPJ Regen Med. 2022 Jan 12;7:3. doi: 10.1038/s41536-021-00196-2 (PMC8755719; doi:10.1038/s41536-021-00196-2)
Supplement: Supplementary file 3 — Reporting Summary [file 41536_2021_196_MOESM3_ESM.pdf]

nature portfolio

Corresponding author(s): Yana Zavros

Last updated by author(s): Sep 10, 2021

Reporting Summary

Nature Portfolio wishes to improve the reproducibility of the work that we publish. This form provides structure for consistency and transparency in reporting. For further information on Nature Portfolio policies, see our Editorial Policies and the Editorial Policy Checklist.

Statistics

For all statistical analyses, confirm that the following items are present in the figure legend, table legend, main text, or Methods section.

n/a	Confirmed	
☐	☒	The exact sample size (*n*) for each experimental group/condition, given as a discrete number and unit of measurement
☐	☒	A statement on whether measurements were taken from distinct samples or whether the same sample was measured repeatedly
☐	☒	The statistical test(s) used AND whether they are one- or two-sided<br>*Only common tests should be described solely by name; describe more complex techniques in the Methods section.*
☒	☐	A description of all covariates tested
☒	☐	A description of any assumptions or corrections, such as tests of normality and adjustment for multiple comparisons
☐	☒	A full description of the statistical parameters including central tendency (e.g. means) or other basic estimates (e.g. regression coefficient) AND variation (e.g. standard deviation) or associated estimates of uncertainty (e.g. confidence intervals)
☒	☐	For null hypothesis testing, the test statistic (e.g. *F*, *t*, *r*) with confidence intervals, effect sizes, degrees of freedom and *P* value noted<br>*Give P values as exact values whenever suitable.*
☒	☐	For Bayesian analysis, information on the choice of priors and Markov chain Monte Carlo settings
☒	☐	For hierarchical and complex designs, identification of the appropriate level for tests and full reporting of outcomes
☒	☐	Estimates of effect sizes (e.g. Cohen's *d*, Pearson's *r*), indicating how they were calculated

Our web collection on statistics for biologists contains articles on many of the points above.

Software and code

Policy information about availability of computer code

Data collection	NA
Data analysis	NA

For manuscripts utilizing custom algorithms or software that are central to the research but not yet described in published literature, software must be made available to editors and reviewers. We strongly encourage code deposition in a community repository (e.g. GitHub). See the Nature Portfolio guidelines for submitting code & software for further information.

Data

Policy information about availability of data

All manuscripts must include a data availability statement. This statement should provide the following information, where applicable:

- Accession codes, unique identifiers, or web links for publicly available datasets
- A description of any restrictions on data availability
- For clinical datasets or third party data, please ensure that the statement adheres to our policy

The datasets generated during and/or analyzed during the current study are available in the ReDATA repository, https://data.library.arizona.edu/services/research-data-repository-redata#exceptions. The datasets generated during and/or analyzed during the current study are also available from the corresponding author on reasonable request. All data generated or analyzed during this study are included in this published article.

Field-specific reporting

Please select the one below that is the best fit for your research. If you are not sure, read the appropriate sections before making your selection.

☒ Life sciences ☐ Behavioural & social sciences ☐ Ecological, evolutionary & environmental sciences

For a reference copy of the document with all sections, see nature.com/documents/nr-reporting-summary-flat.pdf

Life sciences study design

All studies must disclose on these points even when the disclosure is negative.

Sample size	Sample size is based on assessment of power analysis using SigmaStat software. Data collected from each study from at least 3-4 in vitro biological replicates were analyzed first by obtaining the mean + standard error of the mean (SEM). Significance of the results will then be tested using commercially available software (GraphPad Prism, GraphPad software, Dan Diego, CA). Sample size analysis showed 6 mice used in these experiments at each time point achieved a 0.8 power.
Data exclusions	No data was excluded
Replication	Experimental/technical replicates were included in the study
Randomization	No relevant to the study
Blinding	Investigators were blinded to the study during quantification of the data.

Reporting for specific materials, systems and methods

We require information from authors about some types of materials, experimental systems and methods used in many studies. Here, indicate whether each material, system or method listed is relevant to your study. If you are not sure if a list item applies to your research, read the appropriate section before selecting a response.

Materials & experimental systems

n/a	Involved in the study	
☐	☒	Antibodies
☒	☐	Eukaryotic cell lines
☒	☐	Palaeontology and archaeology
☐	☒	Animals and other organisms
☐	☒	Human research participants
☒	☐	Clinical data
☒	☐	Dual use research of concern

Methods

n/a	Involved in the study	
☒	☐	ChIP-seq
☐	☒	Flow cytometry
☒	☐	MRI-based neuroimaging

Antibodies

Antibodies used	The following primary antibodies and dilutions were used: 1: 100 mouse anti CCR2 (Novus Biologicls, NBP1-48338), 1:500 mouse anti CD68 (Novus Biologicls, NBP2-37265) slides and whole mount organoids were imaged on a Zeiss LSM710 LIVE Duo Confocal Microscope.<br><br>Flow cytometry: Cells were then incubated with CD16/CD32 FcBlock (BD Biosciences, 553141) for 5 minutes at 4°C. Cells were labeled using a 1:100 concentration of antibodies against APC-conjugated F4/80 (Thermo Fisher Scientific, MF48005), FITC-conjugated CD11b (Thermo Fisher Scientific, RM2801), PE-conjugated Ly6G (BD Biosciences, 551461), and PerCP-Cy5.5-conjugated Ly6C (eBiosciences, 45-5932) for 20 minutes at room temperature in dark and then fixed with fixative medium A (Thermo Fisher Scientific, GAS004) for 15 minutes at room temperature.
Validation	1. Unstained and single-stained cells controls were used as compensation controls for the flow cytometry experiments.<br>2. Key antibodies will be verified using samples known to have the specific antigens, and also samples which are absent of those specific antigens by immunoblotting and/or flow cytometry.

Animals and other organisms

Policy information about studies involving animals; ARRIVE guidelines recommended for reporting animal research

Laboratory animals	1. C57BL/5 Mice<br>2. LysMCre (The Jackson Laboratory, B6.129P2-Lyz2tm1(cre)Ifo/J, stock number 004781) with the Smoflx/flx (The Jackson Laboratory, Smotm2Amc/J, stock number 004526 | Smoc) mice<br>3. 4-8 weeks of age<br>4. Both males and females were included in these studies.
Wild animals	NA
Field-collected samples	NA
Ethics oversight	All mouse studies were approved by the University of Cincinnati Institutional Animal Care and Use Committee (IACUC) that maintains an American Association of Assessment and Accreditation of Laboratory Animal Care (AAALAC) facility (Protocol number 19-571).

Note that full information on the approval of the study protocol must also be provided in the manuscript.

Human research participants

Policy information about studies involving human research participants

Population characteristics	Every patient who agrees to sign the consent is enrolled. Our recruitment criteria include both genders, minorities and all racial and ethnic categories. Human gastric tissue and blood is collected during sleeve gastrectomies with the approval of the Institutional Review Board (University of Cincinnati and 2014-0427, Cincinnati Children's Hospital Medical Center).
Recruitment	Human stomach tissue was collected and de-identified by Dr. Michael Helmrath (co-author) Cincinnati Children's Hospital Medical Center prior to being shipped to Yana Zavros. Patient consent was performed by Dr. Helmrath at Cincinnati Children's Hospital Medical Center.
Ethics oversight	Human fundus/corpus tissue was collected from sleeve gastrectomies (IRB protocol number: 2015-5537, University of Cincinnati and 2014-0427, Cincinnati Children's Hospital Medical Center; IRB protocol number: 1912208231R001, University of Arizona Human Subjects Protection Program)

Note that full information on the approval of the study protocol must also be provided in the manuscript.

Flow Cytometry

Plots

Confirm that:

☒ The axis labels state the marker and fluorochrome used (e.g. CD4-FITC).

☒ The axis scales are clearly visible. Include numbers along axes only for bottom left plot of group (a 'group' is an analysis of identical markers).

☒ All plots are contour plots with outliers or pseudocolor plots.

☒ A numerical value for number of cells or percentage (with statistics) is provided.

Methodology

Sample preparation	Cells were collected from uninured and injured tissue digest, centrifuge at 400 xg for 5 minutes and were resuspended in PBS with 5% bovine serum albumin. Cells were then incubated with CD16/CD32 FcBlock (BD Biosciences, 553141) for 5 minutes at 4°C. Cells were labeled using the antibodies detailed in the methods section of the manuscript.
Instrument	FACS Calibur flow cytometer (BD Biosciences), Cytek Aurora
Software	FlowJo<br>OMIQ
Cell population abundance	Total of 100,000 events were collected
Gating strategy	F480+/CD11b+/Ly6Chi/Ly6Gneg/CCR2+

☒ Tick this box to confirm that a figure exemplifying the gating strategy is provided in the Supplementary Information.
